# Early life adversity shapes social subordination and cell type–specific transcriptomic patterning in the ventral hippocampus

**DOI:** 10.1126/sciadv.adj3793

**Published:** 2023-12-01

**Authors:** Aron Kos, Juan Pablo Lopez, Joeri Bordes, Carlo de Donno, Julien Dine, Elena Brivio, Stoyo Karamihalev, Malte D. Luecken, Suellen Almeida-Correa, Serena Gasperoni, Alec Dick, Lucas Miranda, Maren Büttner, Rainer Stoffel, Cornelia Flachskamm, Fabian J. Theis, Mathias V. Schmidt, Alon Chen

**Affiliations:** ^1^ Department of Stress Neurobiology and Neurogenetics, Max Planck Institute of Psychiatry, Munich, Germany.; ^2^ Department of Neuroscience, Karolinska Institutet, Stockholm, Sweden.; ^3^ Research Group Neurobiology of Stress Resilience, Max Planck Institute of Psychiatry, Munich, Germany.; ^4^ Institute of Computational Biology, Helmholtz Zentrum München, German Research Center for Environmental Health, Neuherberg, Germany.; ^5^ Department of Mathematics, Technische Universität München, Munich, Germany.; ^6^ Department of Brain Sciences, Weizmann Institute of Science, Rehovot, Israel.; ^7^ International Max Planck Research School for Translational Psychiatry (IMPRS-TP), Munich, Germany.; ^8^ Institute of Lung Health and Immunity, Helmholtz Munich, Munich, Germany.; ^9^ Department of Statistical Genetics, Max Planck Institute of Psychiatry, Munich, Germany.; ^10^ Department of Molecular Neuroscience, Weizmann Institute of Science, Rehovot, Israel.

## Abstract

Adverse events in early life can modulate the response to additional stressors later in life and increase the risk of developing psychiatric disorders. The underlying molecular mechanisms responsible for these effects remain unclear. Here, we uncover that early life adversity (ELA) in mice leads to social subordination. Using single-cell RNA sequencing (scRNA-seq), we identified cell type–specific changes in the transcriptional state of glutamatergic and GABAergic neurons in the ventral hippocampus of ELA mice after exposure to acute social stress in adulthood. These findings were reflected by an alteration in excitatory and inhibitory synaptic transmission induced by ELA in response to acute social stress. Finally, enhancing the inhibitory network function through transient diazepam treatment during an early developmental sensitive period reversed the ELA-induced social subordination. Collectively, this study significantly advances our understanding of the molecular, physiological, and behavioral alterations induced by ELA, uncovering a previously unknown cell type–specific vulnerability to ELA.

## Teaser

Early life adversity shapes social status, molecular signatures in the brain, and the response to social stress later in life.

## INTRODUCTION

A large body of evidence has shown that early life abuse, neglect, and other forms of adversity can result in a range of mental health problems such as major depressive disorder, anxiety disorders, bipolar disorder, and personality disorders (*1*–*5*). Furthermore, exposure to additional severe stressors throughout life can dramatically increase the likelihood for developing such disorders (*6*–*9*). Early life adversity (ELA) can affect the neurodevelopmental trajectories and maturation of various cell types within the brain (*10*–*12*). This in turn can disrupt the formation and function of a number of brain regions. For example, imaging studies in humans and mice revealed that the prefrontal cortex (PFC), amygdala, and hippocampus (HPC) are altered in size and function (*13*–*17*). It is thought that these brain regions are susceptible to ELA due to their relatively high abundance of stress hormone receptors, making them particularly sensitive to increased levels of corticosteroids (*18*, *19*). Moreover, in animal models, ELA has been shown to disturb the transcriptional development in various brain regions, resulting in gene expression changes during adulthood (*20*–*22*). Although these previous studies have provided considerable progress, much of the underlying molecular mechanisms of how ELA alters the response to additional stressors later in life remains unclear. An important but currently unknown facet is whether ELA exposure can differentially affect the plethora of cell types found within the brain.

Using single-cell RNA sequencing (scRNA-seq), we set out to identify cell type–specific transcriptomic signatures associated with ELA and, more importantly, whether these signatures are influenced by additional stressors later in life. First, we performed a comprehensive behavioral characterization using a high-throughput automated behavioral tracking system combined with classical tests and found that ELA exposure leads to social subordination in groups of mice. We subsequently exposed adult ELA animals to acute social defeat stress, a type of stressor based on social hierarchy and dominance in mice, and performed scRNA-seq analysis on the ventral HPC (vHPC), a known region susceptible to ELA exposure (*23*, *24*). While other regions, such as the amygdala, the medial PFC (mPFC), and the striatum, are also key areas known to be involved in stress response regulation, the vHPC has been shown to play an important role in emotion and anxiety behaviors (*25*–*28*). In addition, the vHPC has been recently shown to be part of the social dominance circuitry by regulation of social interactions and social memory formation (*29*–*32*). Thus, it appears a particularly relevant region to investigate how ELA influence stress response later in life and, most importantly, its effects on social interaction behaviors and hierarchy formation. The scRNA-seq analysis identified specific effects of ELA across multiple cell types, including neurons, glia, and vascular cells. Most notably, we found that ELA exposure resulted in a striking change in the transcriptional state of glutamatergic and GABAergic neurons in response to acute social stress. Systematic analyses and characterization of the interactions between ELA and acute social stress in adulthood identified distinct, cell type–specific, and cell cluster–wide transcriptional patterning, uncovering acute social stress–regulated gene pathways susceptible to ELA exposure. Electrophysiological recordings in acute vHPC slices revealed that ELA disrupts excitation and inhibition transmission in response to acute social stress. Increasing inhibitory tone through transient diazepam treatment during a critical period resulted in a reversal of the ELA-induced social subordination behaviors. Together, our findings show that ELA can shape the acute stress response by differentially affecting glutamatergic and GABAergic neural networks within the vHPC, uncovering a cell type–specific mechanism for ELA vulnerability.

## RESULTS

### ELA leads to social subordination

**Fig. 1.**
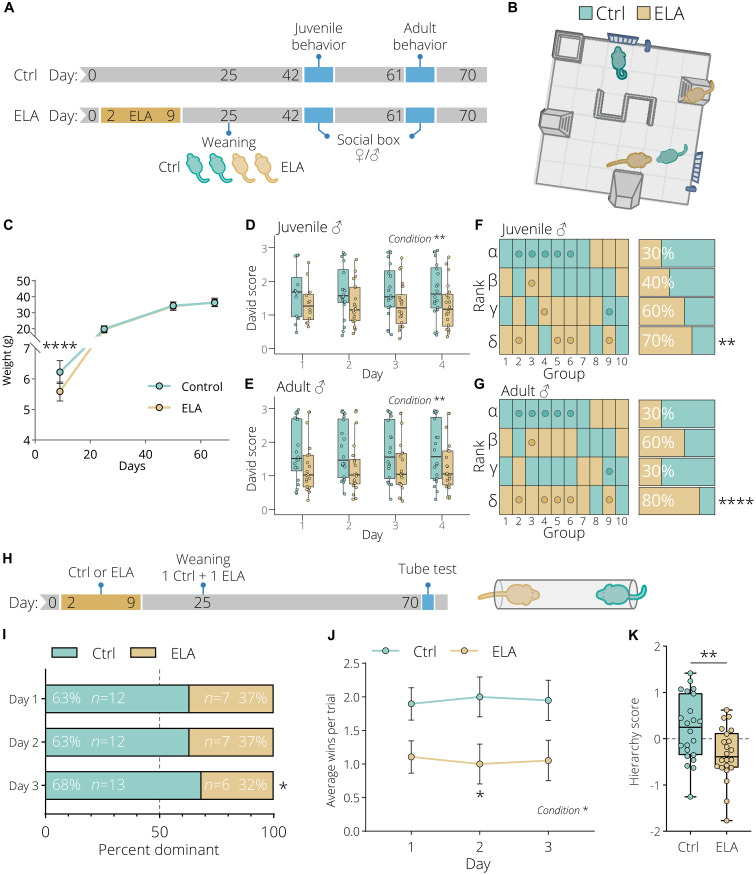
Early life adversity leads to social subordination in group-housed animals. (**A**) Experimental timeline of control and early life adversity (ELA) animals. Animals were weaned in groups of four consisting of two control (light green) and two ELA (light gold) animals. Ten groups were behaviorally tested in the social box (SB) at the juvenile (~6 weeks) and adult (~9 weeks) stage. (**B**) The 60 cm × 60 cm SB arena containing an s-wall, nest, small nest, two feeders, and two ramps. (**C**) Reduced weight gain of ELA animals compared to control animals. Data represent mean ± SD, *n* = 20 per condition. Two-way repeated-measures analysis of variance (ANOVA) with Bonferroni post hoc test. ELA-exposed animals have a lower daily David score based on chases at the (**D**) juvenile and (**E**) adult stage. Two-way repeated-measures ANOVA. (**F** and **G**) Hierarchy distribution based on the cumulative David score over 4 days of all 10 groups tested in the SB. The hierarchy order is from alpha, beta, gamma to sigma, with the highest-ranking animal being the alpha. Mice selected for sequencing are highlighted with a dot. Both at the (F) juvenile and (G) adult stage, ELA animals display a significantly lower social rank. Yates’ corrected chi-square test. (**H**) Experimental timeline of animals tested in the tube test. Animals were pair-weaned with one control and one ELA animal. (**I**) Hierarchy distribution over the three tube test days. Yates’ corrected chi-square test. (**J**) ELA exposure significantly reduced the average number daily tube test wins. Data represent mean ± SEM. Two-way repeated-measures ANOVA with Bonferroni post hoc test. (**K**) ELA animals have a significantly lower hierarchy score. Box plots represent the 25%, median, and 75% quartile; whiskers span 1.5 × interquartile range (IQR). Unpaired *t* tests, two-tailed. *P < 0.05, **P < 0.01, ****P < 0.0001.

The well-established limited bedding and nesting model leads to fragmented maternal care, resulting in long-term physiological alterations in mice (*20*, *33*, *34*). We used this ELA paradigm to study the impact of chronic stress during a critical developmental period in mice ranging from postnatal day (PND) 2 to 9 (Fig. 1A). Both female and male mice were group-housed (separately), pairing two control and two ELA-exposed animals followed by in-depth behavioral characterization during adolescence and adulthood using the social box (SB) (*35*). The SB is a semi-enriched, semi-naturalistic living environment, which enables automatic location tracking and behavioral labeling of group-housed animals over multiple consecutive days (Fig. 1B). At 9 days of age, ELA-exposed animals showed a significant reduction in body weight compared to control animals, while no weight differences were observed past day 25 (Fig. 1C). Continuous automated behavior tracking in the SB resulted in a total of 58 trajectory-based readouts collected over four consecutive days at the juvenile and adult stage from male and female mice. Only one individual readout was found to be significantly different in male juvenile ELA animals compared to control animals, while in adulthood a greater number of behavioral differences were observed (fig. S1, A to F). Similarly, in female mice, at the juvenile stage, no behavioral differences were observed, while in adult animals several behaviors were found to be significantly altered (fig. S2, A to F). However, this is expected as the establishment of social hierarchies in groups of mice becomes more stable during early adulthood (*36*). Principal components analysis (PCA) using all 58 readouts resulted in a significant separation between the male control and ELA condition in PC1 for both juvenile and adult animals (fig. S1, C and D). The same analysis in female mice revealed no significant separation (fig. S2, C and D). This observation indicates that ELA induces a set of stable behavioral alterations in male mice, which is quantifiable with SBs, and persists over an extended period of time. In both juvenile and adult animals, the top loadings with the greatest contribution to the variation in PC1 were almost exclusively pairwise, indicating a change in social interactions (figs. S1, E and F, and S2, E and F). Since social hierarchy is typically a stable behavioral feature that has a powerful influence on social dynamics, we further explored whether ELA can influence the hierarchy of groups of mice. Social dominance in the SB was calculated using the well-established David’s score (DS) method (*37*). This analysis revealed that both male juvenile and adult ELA mice displayed a significantly lower DS (Fig. 1, D and E) and have an overall lower social rank determined using the cumulative DS (Fig. 1, F and G). In female mice, we observed no differences in juvenile mice, while adult female ELA mice displayed a mild but significantly lower DS and a lower social rank compared to control animals (fig. S2, G to J). Our data indicate that the effect of ELA on social rank is stronger in male mice. Furthermore, male mice have been shown to form more despotic and steeper hierarchies (*38*, *39*). We therefore decided to continue to explore the effects of ELA on hierarchies in male mice only. In an effort to replicate these findings, we generated a separate cohort of male mice in which we paired one control and one ELA animal (Fig. 1H). The hierarchy of these pair-housed animals was subsequently tested in the tube test, a classical and validated social dominance task (*40*). This task was performed for three consecutive days, with the animal that is pushing the other animal out of the tube (called a “win”) being considered the dominant mouse. Consistent with our findings in the SB, ELA mice were significantly less dominant and had fewer average wins in the tube test (Fig. 1, I and J). Furthermore, by calculating a *z*-score, we determined the hierarchy of a pair of mice, integrating the average number of wins, pushes, and percent time spent retreating and moving forward (fig. S3). This score was significantly lower in ELA-exposed animals compared to control animals (Fig. 1K). Collectively, these results revealed that ELA exposure leads to social subordination using both classical and high-throughput behavioral paradigms.

### ELA leads to cell type–specific changes of transcriptional states in the vHPC

**Fig. 2.**
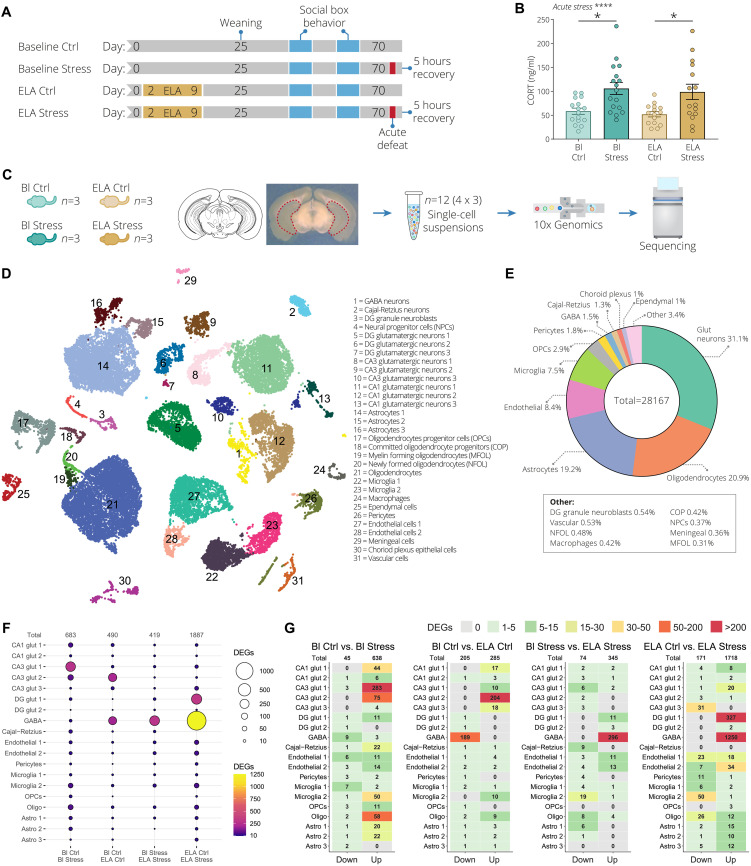
ELA blunts the acute stress–induced transcriptional activation in ventral hippocampal CA3 glutamatergic neurons. (**A**) Experimental timeline. “Baseline” indicates animals that were not exposed to ELA. The blue sections indicate when animals were behaviorally tested in the SB. Half of the baseline and half of the ELA animals underwent acute social defeat stress (red blocks). The four conditions baseline control (Bl Ctrl), baseline stress (Bl Stress), ELA control (ELA Ctrl), and ELA stress were characterized using single-cell RNA sequencing (scRNA-seq). (**B**) Corticosterone (CORT) levels 5 hours after acute social defeat exposure. Data represent mean ± SEM. Two-way ANOVA with Tukey post hoc test. (**C**) Outline of the scRNA-seq experiment. For each condition, three animals (total of 12 individual samples) were used from which the ventral hippocampus (vHPC) was bilaterally isolated followed by scRNA-seq. (**D**) Cell clustering of all 12 mouse vHPC samples depicted using uniform manifold approximation and projection (UMAP). The colors represent the 31 Louvain groups of individual cell types. (**E**) Distribution of all major cell types identified in the vHPC, with a total of 28,167 collected cells. A number of cell types are abbreviated as follows: glutamatergic neurons (Glut neurons), oligodendrocyte progenitor cells (OPCs), GABAergic neurons (GABA), newly formed oligodendrocytes (NFOL), committed oligodendrocyte progenitors (COP), neural progenitor cells (NPCs), and myelin-forming oligodendrocytes (MFOL). (**F**) Dot plot of the number of differentially expressed genes (DEGs). From left to right: baseline control versus baseline stress, baseline control versus ELA control, baseline stress versus ELA stress, and ELA control versus ELA stress. The size and color of the circle indicate the number of DEGs. (**G**) Identified DEGs in 19 clusters split by down- and up-regulated. The number in each tile indicates the number of DEGs. *P < 0.05, ****P < 0.0001.

In an effort to understand how ELA affects the response to an acute social stressor in adulthood, we performed an in-depth cell type–specific molecular characterization using scRNA-seq. Following ELA and SB behavioral phenotyping, adult animals (~PND 70) were exposed to acute social defeat stress, a type of stressor based on dominance, which is an important behavior exhibited by mice to establish and maintain social hierarchies (*41*, *42*). Half of the control and half of the ELA animals were exposed to acute social defeat stress (Fig. 2A). Animals not exposed to ELA were labeled as “baseline,” while the acute social defeat stressed condition was labeled as “stress.” This generated the following four conditions: baseline control (Bl Ctrl), baseline stress (Bl Stress), ELA control (ELA Ctrl), and ELA stress (Fig. 2A). Tissue was collected 5 hours after acute social defeat stress to capture the second wave of transcription initiated by immediate early genes (*43*–*46*). The selection of this time point allows the exploration of important downstream effectors of stress and avoids potential confounding effects derived from the immediate early gene responses. Plasma corticosterone (CORT) levels were significantly increased in baseline and ELA animals 5 hours after acute social defeat stress, verifying that this paradigm leads to a robust stress response (Fig. 2B). The vHPC was selected for molecular analysis as it is a well-known region for the control of anxiety-like behaviors, and more recently was shown to be critically involved in social interaction behaviors and social memory formation, key aspects for establishing and maintaining social hierarchies (*29*–*32*). Three mice from each group were selected for the four conditions of our scRNA-seq analysis. Here, by “group,” we refer to mice that share the same home cage and SB (each group consisting of two control-reared and two ELA-exposed mice). The selection of mice was based on the PCA analysis of the SB behavior data (highlighted in fig. S1, C and D), generating a total of 12 scRNA-seq samples (Fig. 2C).

After scRNA-seq, we analyzed the resulting data using Scanpy, following established best practices (*47*). After quality control (QC) analysis, we clustered cells using graph-based methodologies (Louvain) (*48*). Uniform manifold approximation and projection (UMAP) plots were used to visualize the clusters (*49*). We identified a total of 31 distinct clusters and determined the cell identity based on well-established cell type–specific markers from literature (Fig. 2D and fig. S4A) (*50*). Furthermore, nine of the glutamatergic neuron clusters were localized to hippocampal subregions (CA1, CA3, and DG) based on region-specific gene markers (fig. S4, A to C) (*51*, *52*). The largest cell clusters consisted of glutamatergic neurons (31.1%), oligodendrocytes (20.9%), astrocytes (19.2%), endothelial cells (8.4%), and microglia cells (7.5%) (Fig. 2E). The remaining 14 smaller clusters (<5%) constituted 12.9% of the total 28,167 cells. On the basis of our QC metrics, no significant changes were found in cell type composition (fig. S5, A to C), proportions (fig. S6, A and B), or distribution per sample (fig. S7). Subsequently, we performed differential expression analysis using diffxpy (*53*). We further explored the identified differentially expressed genes (DEGs) of a subset of 19 cell clusters containing at least 300 cells (25 cells per sample) or more. This analysis was done pairwise, comparing baseline control with baseline stress (Bl Ctrl versus Bl Stress), baseline control with ELA control (Bl Ctrl versus ELA Ctrl), baseline stress with ELA stress (Bl Stress versus ELA Stress), and ELA control with ELA stress (ELA Ctrl versus ELA Stress). We found that acute stress (Bl Ctrl versus Bl Stress) resulted in a total of 683 DEGs [log_2_ fold change (log_2_FC) |0.5| and *q* < 0.05], with the largest fraction of DEGs originating from glutamatergic neurons in CA3 and CA1 (Fig. 2F). Acute stress also induced a substantial number of DEGs in oligodendrocyte and microglia cell clusters. Furthermore, acute stress exposure resulted in almost exclusively up-regulated genes (Fig. 2G). ELA dysregulated the expression of 490 genes compared to controls (Bl Ctrl versus ELA Ctrl), with most genes altered in CA3 glutamatergic cluster 2 and GABAergic neurons (Fig. 2F). Moreover, the DEGs in the CA3 glutamatergic cluster 2 were exclusively up-regulated, while in the GABAergic neurons, genes were exclusively down-regulated (Fig. 2G). ELA with the acute stress background (Bl Stress versus ELA Stress) resulted in a total of 419 DEGs, of which 296 exclusively up-regulated DEGs were detected in GABAergic neuron cluster (Fig. 2, F and G). Acute stress exposure with an ELA background (ELA Ctrl versus ELA Stress) dysregulated 1887 genes. Interestingly, and in contrast with our acute stress condition (without ELA), we found a particularly strong transcriptional up-regulation in GABAergic neurons (Fig. 2, F and G). Additionally, the robust transcriptional activation of the CA1 and CA3 glutamatergic neurons we observed in the Bl Ctrl versus Bl Stress condition was almost completely blunted in the ELA Ctrl versus ELA Stress condition. This suggests that ELA alters the transcriptional state of GABAergic and glutamatergic neurons in response to acute social defeat stress. We then compared the overlap between gene sets by combining all unique DEGs from the top eight clusters in terms of number of DEGs. Here, we found that most DEGs were unique to each cluster, displaying overall a minimal degree of overlap (fig. S8). The largest similarity was observed between the GABA and dentate gyrus (DG) glut 1 clusters, with 148 overlapping genes. Among these eight clusters, a relatively minimal total number of 44 genes were differentially expressed in more than three clusters (fig. S8). Together, our results show that stress-induced transcriptional changes are largely cell type–specific and that ELA leads to an altered transcriptional state in glutamatergic and GABAergic neurons in response to acute social stress.

### Social stress leads to cell type–specific transcription patterning by ELA

**Fig. 3.**
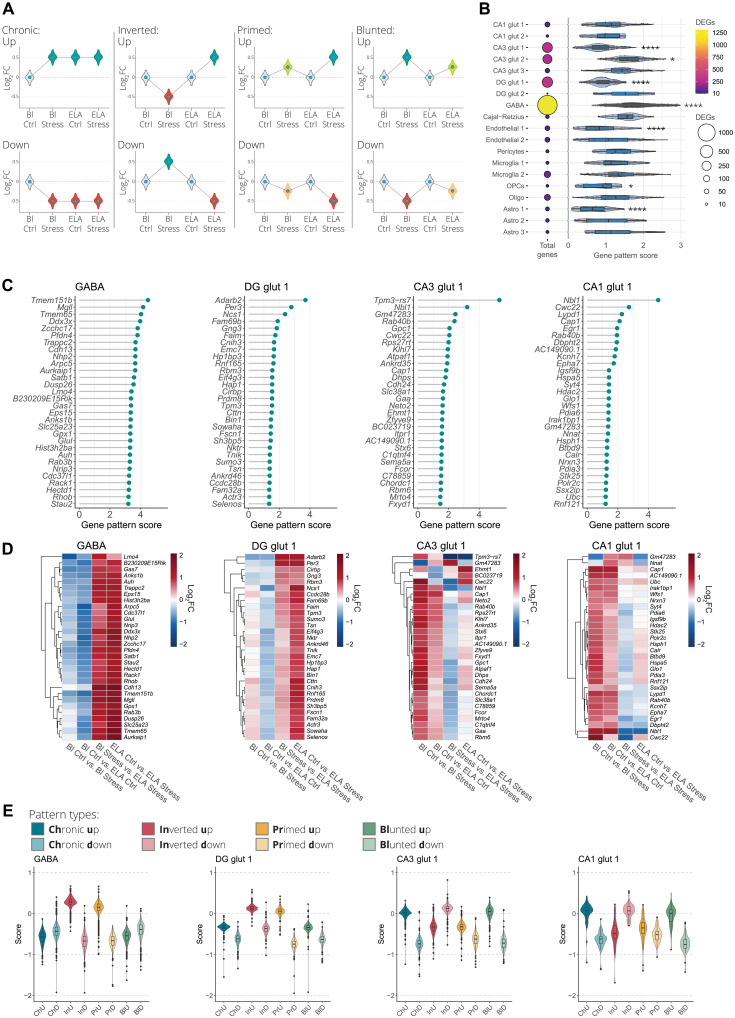
Widespread and cell type–specific transcriptional patterning induced by ELA. (**A**) Illustration of the theoretical transcriptional patterning effects where ELA interacts with gene expression changes induced by acute stress exposure. The transcriptional patterning effects are categorized as up- or down-regulated chronic, inverted, primed, or blunted expression. (**B**) Dot plot on the left side represents all combined DEGs in 19 clusters identified in all four comparisons (Bl Ctrl versus Bl Stress, Bl Ctrl versus ELA Ctrl, Bl Stress versus ELA Stress, and ELA Ctrl versus ELA Stress). Size and the color of the circles indicate the number of DEGs. The violin plot on the right side indicates the calculated gene patterning score for each DEG associated with each cluster. The gene patterning score of several clusters significantly deviates from the collection of all gene patterning scores from all 19 clusters. The higher the score, the higher the likelihood to find one of the four gene patterning types, while a lower score indicates a lower interaction between ELA and the acute stress. One-way ANOVA with Tukey post hoc test. (**C**) Lollipop plot ranking the top 30 highest scoring genes in GABAergic neurons (GABA), dentate gyrus (DG) glutamatergic neuron cluster 1 (DG glut 1), CA3 glutamatergic neuron cluster 1 (CA3 glut 1), and CA1 glutamatergic neuron cluster 1 (CA1 glut 1). (**D**) Clustered heatmap visualization of the log_2_FC of the top 30 highest scoring genes in the four selected clusters. The four rows in each heatmap represent the four differential expression comparisons. (**E**) Quantification of each of the individual transcriptional patterning types in the four selected clusters identifies distinct transcriptional patterns. Box plots represent the 25%, median, and 75% quartile; whiskers span 1.5 × IQR. *P < 0.05, ****P < 0.0001.

**Fig. 4.**
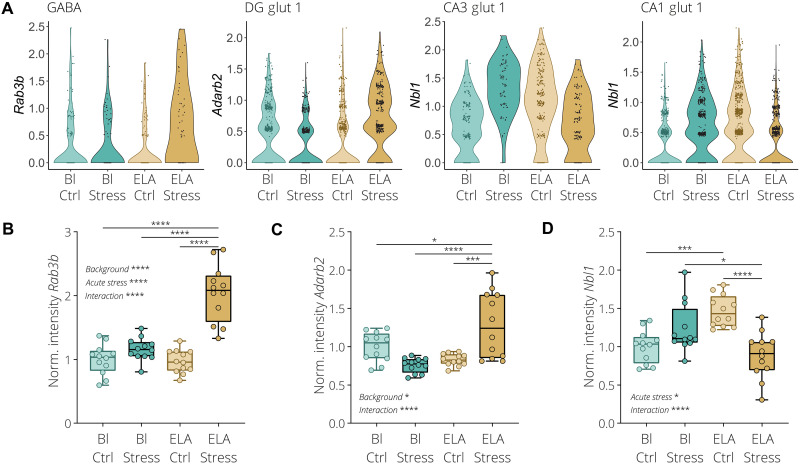
*Rab3b*, *Adarb2*, and *Nbl1* differentially respond to acute stress after ELA exposure. (**A**) Violin plots illustrating the expression of selected top scoring genes determined by scRNA-seq, namely, *Rab3b* in the GABAergic neuron cluster, *Adarb2* in the DG glutamatergic neuron cluster 1, and *Nbl1* in the CA3 glutamatergic neuron cluster 1 and CA1 glutamatergic neuron cluster 1. (**B**) Quantification of the *Rab3b* mRNA expression signal in cells positive for the GABAergic neuron marker *Slc32a1* in the CA3 vHPC region. (**C**) Quantification of the *Adarb2* mRNA expression signal in cells positive for the glutamatergic neuron marker *Slc17a1* in the DG vHPC region. (**D**) Quantification of the *Nbl1* mRNA expression signal in cells positive for the glutamatergic neuron marker *Slc17a1* in the CA3 vHPC region. Box plots represent the 25%, median, and 75% quartile; whiskers span minimum to maximum. Two-way ANOVA with Tukey post hoc test. *P < 0.05, ***P < 0.001, ****P < 0.0001.

To further explore the interaction between ELA and acute social stress in adulthood, we set out to categorize the various transcriptional patterns into four major theoretical types, namely, the up- or down-regulated (i) chronic, (ii) inverted, (iii) primed, or (iv) blunted transcription patterns (Fig. 3A). We devised a scoring method allowing for the systematic detection of genes that display a high degree of interaction between ELA and acute stress. To do so, we combined all unique DEGs from each of the different cell clusters identified in the four comparisons and subsequently calculated the gene pattern score for each gene. A higher score indicates an increased likelihood for detecting one of the aforementioned transcriptional patterning effects. This revealed that the GABA and CA3 glut 2 clusters showed an overall higher degree of gene patterning, while the CA3 glut 1, DG glut 1, endothelial 1, oligodendrocyte precursor cell (OPC), and astrocyte 1 clusters had a lower degree of gene patterning (Fig. 3B). Next, we selected the highest scoring GABAergic neuron cluster that contained the highest number of DEGs together with three of the primary glutamatergic neuron clusters from three hippocampal subregions, namely, the DG glut 1, CA3 glut 1, and CA1 glut 1 clusters. Hierarchical clustering of the top 30 highest scoring genes from the four selected cell clusters indicated that most of these genes display a similar transcriptional patterning type (Fig. 3, C and D). The top scoring genes in the GABA and DG glut 1 clusters tend to be down-regulated or unchanged by acute stress or ELA exposure alone (Bl Ctrl versus Bl Stress and Bl Ctrl versus ELA Ctrl), while combining both acute stress and ELA results in a tendency for these same genes to increase in expression (Bl Stress versus ELA Stress and ELA Ctrl versus ELA Stress) (Fig. 3D). The opposite effect is observed in the CA3 glut 1 and CA1 glut 1 clusters. We expected that genes with a low gene patterning score primarily consist of expression patterns where there is a little interaction between acute stress and ELA (fig. S9A). The 30 lowest scoring genes of the GABA, DG glut 1, CA3 glut 1, and CA1 glut 1 clusters displayed little interaction between ELA and acute stress, where genes were up-regulated to a similar extent both by acute stress alone or combined with the ELA background (fig. S9, B to D). To further characterize the transcriptional patterning, we calculated a score for each of the individual gene pattern types, namely, chronic, inverted, primed, or blunted gene expression patterns (Fig. 3E), where a high score increases the likelihood that one of the patterning types is expressed with the lowest possible score being zero. This revealed that within the GABA and DG glut 1 clusters, the two highest patterning types were inverted up-regulated and primed up-regulated (Fig. 3E). In CA3 glut 1 and CA1 glut 1, chronic up-regulated, inverted down-regulated, and blunted up-regulated patterns had the highest score. Furthermore, the observation that most DEGs display a similar patterning type indicates that these expression patterns tend to occur cell cluster wide. As a proof of principle, we selected high scoring genes from the GABA, DG glut 1, CA3 glut 1, and CA1 glut 1 clusters in an effort to validate our scRNA-seq data and gene patterning detection methods. For the GABA cluster, we selected Ras-related protein Rab-3B (*Rab3b*), a gene exclusively expressed in GABAergic neurons (table S1). According to our analysis, this gene is primed by ELA exposure, resulting in a stronger up-regulation when exposed to acute stress (Fig. 4A). Adenosine deaminase RNA specific B2 (*Adarb2*) was selected from the DG glut 1 cluster, which had the highest score and is specifically expressed in glutamatergic neurons from the DG (table S1). *Adarb2* showed a combination of a priming and inverted transcriptional effect (Fig. 4A). Last, for the CA3 glut 1 and CA1 glut 1 clusters, the neuroblastoma suppressor of tumorigenicity 1 (*Nbl1*) gene was selected for validation after showing the highest score in both clusters. This gene has a high expression in all glutamatergic neurons throughout the vHPC (table S1) and displays a combination of the chronic and inverted transcriptional pattern (Fig. 4A). Using RNAscope fluorescence in situ hybridization, we were able to validate the primed expression of *Rab3b* in GABAergic neurons within the CA3 region of the vHPC, using coexpression with the solute carrier family 32 member 1 (*Slc32a1*) gene (Fig. 4B and fig. S10A). In addition, *Adarb2* was found to be exclusively expressed in glutamatergic neurons of the DG, using coexpression with the solute carrier family 17 member 7 (*Slc17a7*) gene (fig. S10B). Furthermore, *Adarb2* showed a combination of the inverted and primed expression pattern (Fig. 4C). Last, *Nbl1* displayed a similar expression pattern as observed in the scRNA-seq data, with an inverted expression pattern in CA3 glutamatergic neurons (Fig. 4D and fig. S10C). Collectively, our results show that gene patterning is a widespread cell type–specific phenomenon, revealing a high degree of interaction between ELA and acute stress. Furthermore, the relatively high degree of gene patterning detected in GABAergic neurons of the vHPC indicates that within these cells the transcriptional response to acute stress is especially vulnerable to disruption by ELA exposure.

### ELA disrupts stress-induced glutamatergic and GABAergic signal transmission in the vHPC

To explore the functional consequences of the transcriptional dysregulation of acute stress response by ELA exposure, we assessed the biological relationships between the identified DEGs. We used the Metascape gene annotation and functional enrichment tool, which allows for network visualization of similarities between the identified enriched gene clusters (*54*), and analyzed the GABAergic cell cluster since it contained the largest number of DEGs. Using the gene patterning scores associated with each gene as overlay, we identified functional gene clusters that are resilient or susceptible to ELA disruption. We subdivided sets of DEGs in high scoring (top 25%), low scoring (lowest 25%), and medium scoring (middle 50%) genes. This identified that ELA exposure shapes vulnerability of acute stress–activated gene networks in GABAergic neurons particularly involved in cellular metabolism and synaptic function (fig. S11, A and B).

**Fig. 5.**
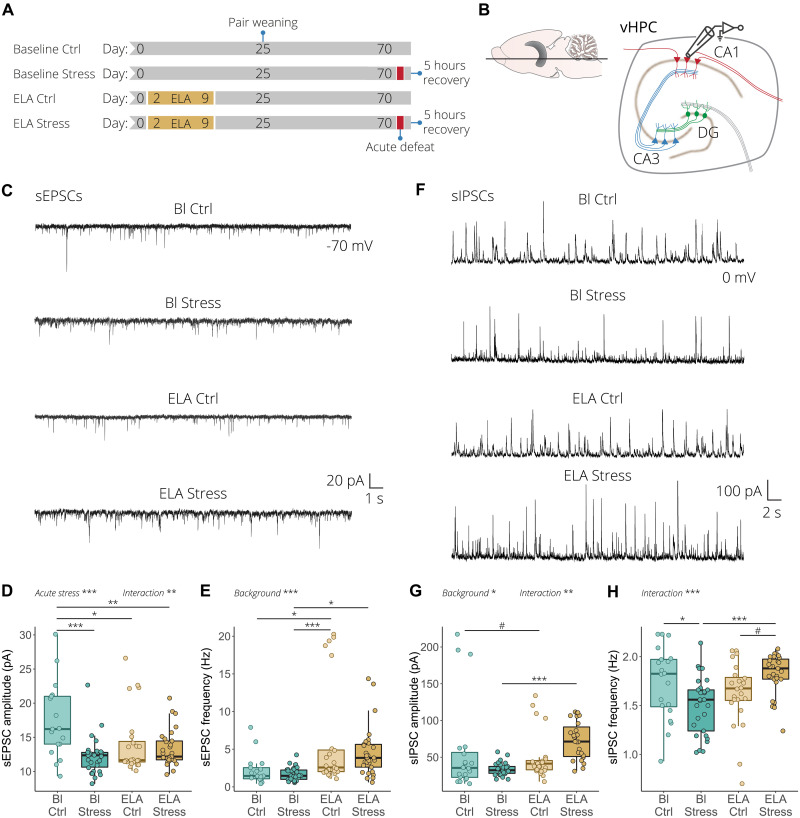
ELA alters excitation and inhibition transmission in response to acute stress in the vHPC. (**A**) Experimental timeline for the electrophysiological recording of the baseline control, baseline stress, ELA control, and ELA stress conditions. (**B**) Illustration of an ex vivo acute ventral hippocampal brain slice depicting whole-cell patch-clamp recording obtained from CA1 pyramidal neurons. (**C**) Representative spontaneous excitatory postsynaptic current (sEPSC) recordings from CA1 pyramidal neurons. (**D**) Quantification of sEPSC amplitude. Acute stress reduces sEPSC amplitude, while ELA-exposed animals have chronically reduced sEPSC amplitude with no further effect of acute stress (left). (**E**) In wild-type mice, acute stress has no effect on sEPSC frequency, while in ELA mice it increases sEPSC frequency (right). (**F**) Representative spontaneous inhibitory postsynaptic current (sIPSC) recordings from CA1 pyramidal neurons. (**G**) Quantification of sIPSC amplitude showed that acute stress had no effect in wild-type animals, while acute stress significantly increased sIPSC amplitude in mice with ELA background (left). (**H**) Acute stress significantly reduced sIPSC frequency in wild-type mice, while the frequency was increased in response to acute stress in ELA mice (right). sEPSCs and sIPSCs were recorded at –70 and 0 mV, respectively, from *n* = 17 to 30 cells/four to six mice per group. Box plots represent the 25%, median, and 75% quartile; whiskers span 1.5 × IQR. Two-way ANOVA with Tukey post hoc test. ^#^*P* < 0.1, *P < 0.05, **P < 0.01, ***P < 0.001.

Considering these results, combined with our scRNA-seq results showing that ELA alters the transcriptional state of glutamatergic and GABAergic neurons, we decided to investigate whether this correlates with electrophysiological changes in the vHPC neuronal network by measuring spontaneous excitatory and inhibitory postsynaptic currents (sEPSCs and sIPSCs) in an independent cohort of mice (Fig. 5A). Both sEPSCs and sIPSCs were measured from the same visually identified CA1 pyramidal neurons in acute vHPC slices at –70 and 0 mV, respectively (Fig. 5B). Concerning the glutamatergic transmission, in wild-type mice, acute stress led to a significant reduction in the amplitude without affecting the frequency of the sEPSCs. On the other hand, in mice subjected to ELA, the response to acute stress was significantly disrupted. The amplitude of sEPSCs was significantly decreased, while the frequency was chronically elevated, with no significant effect of acute stress exposure (Fig. 5, C to E). Regarding the inhibitory transmission, in wild-type mice, acute stress did not affect the amplitude of sIPSC but significantly decreased their frequency (Fig. 5, F to H). In ELA mice, acute stress resulted in an increase in the amplitude compared to wild-type mice exposed to acute stress. Finally, contrary to what was observed in wild-type animals, acute stress increased the frequency of sIPSCs in mice with ELA history (Fig. 5, F to H). Together, these results suggest that ELA exposure significantly alters the excitatory and inhibitory signal transmission in the vHPC in response to an acute social stress. This indicates that the ELA-induced transcriptional changes observed in glutamatergic and GABAergic neurons correlate with functional changes in network activity in the vHPC. This may suggest that the ELA-induced transcriptional changes observed in glutamatergic and GABAergic neurons translate to functional changes in network activity.

### Transient early life diazepam treatment reversed the ELA subordination phenotype

**Fig. 6.**
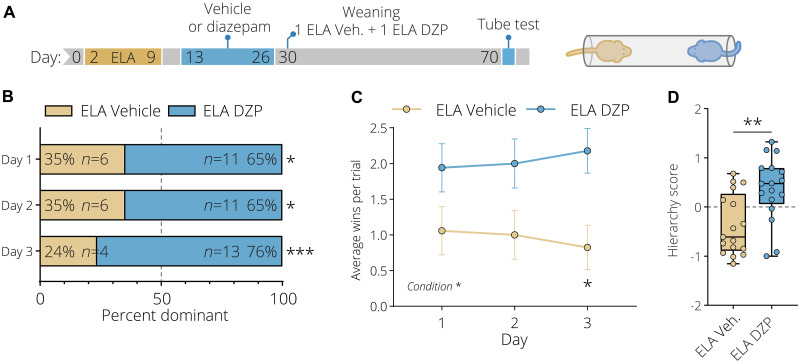
Diazepam reverses the ELA-induced subordination behaviors. (**A**) Experimental timeline of the early life chronic diazepam treatment of ELA-exposed animals. Animals were pair-housed after the chronic diazepam treatment, with each pair consisting of one ELA saline–treated (light gold) and one ELA diazepam–treated (blue) animal. The hierarchy of the pairs was subsequently tested in the tube test in adulthood. (**B**) Hierarchy distribution over the three tube test days shows that diazepam-treated ELA animals are significantly more dominant over ELA saline–treated animals. Yates’ corrected chi-square test. (**C**) Diazepam treatment of ELA animals significantly increased the average number of daily tube test wins. Data represent mean ± SEM. Two-way repeated-measures ANOVA with Bonferroni post hoc test. (**D**) Diazepam-treated ELA animals have a significantly higher hierarchy score combining the average number of wins, head pushes, and the time they spent retreating and moving forward. Box plots represent the 25%, median, and 75% quartile; whiskers span minimum to maximum. Unpaired *t* tests, two-tailed. *P < 0.05, **P < 0.01, ***P < 0.001.

It has previously been shown that systemic administration of benzodiazepine over multiple days early in life can trigger a critical period of increased synaptic plasticity (*55*, *56*). Furthermore, we identified that ELA leads to social subordination and results in robust transcriptional activation of GABAergic neurons. Moreover, ELA affects excitatory and inhibitory signal transmission in response to acute social stress. To investigate the relationship between these observations, we hypothesized that an early life increase in GABAergic transmission in ELA-exposed mice may influence the social subordination phenotype in adulthood. To this end, we treated ELA animals for a sustained period during an early time window ranging from PND 13 until PND 27 with diazepam, a positive allosteric modulator of the GABA type A receptors (20 mg/kg, intraperitoneally, daily), or with a vehicle control solution (Fig. 6A). Mice were subsequently weaned in pairs, consisting of one vehicle-treated animal and one diazepam-treated ELA animal; following this, the social hierarchy was assessed in adulthood using the tube test. ELA mice treated with diazepam were significantly more dominant over vehicle-injected ELA animals, with a significantly higher number of daily average wins (Fig. 6, B and C). Moreover, diazepam-treated ELA animals pushed more, spent more time moving forward, and retreated less compared to control animals (fig. S12). This resulted in a significantly higher composite hierarchy score (Fig. 6D). Collectively, these results show that a transient increase of inhibitory transmission early in life enhances social dominance of ELA mice, revealing that pharmacological intervention can long-lastingly reverse this decisive ELA-induced phenotype.

## DISCUSSION

ELA exposure during critical developmental periods can have profound and lifelong mental health implications. While in the past decade extensive efforts have been made to uncover how ELA conveys susceptibility to psychiatric disorders, many aspects of the molecular underpinnings remain unclear. In this study, we describe a previously unknown cell type–specific vulnerability to ELA exposure, which affects social dominance and the response to social stress later in life. Understanding the molecular and functional mechanisms enabled us to successfully reverse this detrimental phenotype through post hoc pharmacological intervention.

Although the effects of ELA on social behaviors remain relatively unexplored, our findings are in line with a recent study showing that ELA animals displayed increased subordinate behaviors and faster resolution of social conflicts when paired with an unfamiliar conspecific (*57*). This indicates that ELA exposure might affect the early stages of hierarchy formation, leading to stable lifelong alterations in social dominance. The vHPC has been shown to be an important site for the control of emotional and anxiety behaviors (*25*–*28*). Moreover, a number of recent studies have shown the importance of this region in social interaction behaviors (*29*), discrimination of social stimuli (*58*, *59*), and social memory formation (*30*, *31*). These social behaviors play a critical role in the initial stages of social hierarchy formation. Additionally, previous work has identified the vHPC as one of the regions vulnerable to ELA exposure leading to molecular, cellular, and functional changes (*16*, *24*, *60*). Social defeat is a typical dominance behavior displayed in male mice by which they establish and maintain their social rank within a territory. Here, we used scRNA-seq to extract cell type–specific molecular information to study the impact of ELA on the transcriptional response to acute social defeat stress in the vHPC. Through the use of scRNA-seq and subsequent electrophysiological measurements, we revealed that ELA exposure leads to widespread and robust changes in transcriptional patterning in GABAergic and glutamatergic neurons and altered neurophysiological responses to acute social defeat stress in the vHPC. These results indicate that the ELA-induced transcriptional patterning shapes the acute social stress response changing the outcome of these stressful experiences. It is possible that during the early stages of stable hierarchy formation, ELA-exposed mice are more vulnerable to the social defeat they experience by group members, ultimately leading to their subordinate social status.

Although the observed transcriptomic changes are unlikely to be explained by a single gene due to the size and extent of the transcriptomic changes, whereby hundreds of genes were dysregulated over multiple cell types, we selected three genes to validate both our scRNA-seq results and the transcriptomic patterning detection method. Namely, *Rab3b*, *Adarb2*, and *Nbl1* were selected as a proof of concept. RAB3B is exclusively expressed in GABAergic neurons and is a synaptic vesicle protein required for long-term depression of hippocampal inhibitory synapses (*61*). *Adar2b* is part of a family of RNA editing genes and thought to be a dominant-negative regulator of adenosine-to-inosine RNA editing (*62*). Previously, *Adar2b* was shown to be important for hippocampal-dependent fear memory formation (*63*). The bone morphogenetic protein antagonist *Nbl1* plays a role in the development of multiple tissues including the forebrain and has been associated with neuroblastoma formation (*64*–*66*). Although we found it to be abundantly expressed in multiple glutamatergic neuronal populations within the vHPC, its exact function within these cells remains unclear. All three genes were found to have a high degree of transcriptomic patterning according to our scRNA-seq analysis, indicating a strong interaction between ELA and acute social stress later in life. Besides their cell type–specific expression, we were also able to validate the ELA -induced transcriptional patterning of these genes using RNAscope in situ hybridization.

Our findings also revealed that ELA alters vHPC excitation and inhibition transmission and that in particular GABAergic neurons are transcriptionally activated in response to acute social stress. GABAergic neurons have previously been implicated to be altered by ELA exposure. In a recent study, it was shown that maternal separation (MS) with early weaning stress led to changes in neuronal oscillations in the vHPC when exposed to a novel or familiar environment (*24*). Such activity changes are thought to be driven, at least in part, by inhibitory neuronal function. Structural analysis of the vHPC revealed the presence of fewer somatostatin-positive neurons and a disruption of the perineuronal nets of inhibitory neurons in the DG of the vHPC. Recent evidence has shown that ELA exposure can disrupt GABAergic function in the mPFC. Here, ELA through MS resulted in reduced IPSC frequency (*57*). In the amygdala, GABAergic microcircuits were disrupted with both a reduced IPSC frequency and amplitude in rats exposed to MS (*67*). However, none of these studies investigated ELA after exposure to an additional stressor in adulthood. Our results build upon these previous findings and uniquely show that after exposure to ELA, acute social stress (during adulthood) can modulate excitatory and inhibitory transmission in the vHPC. Disruption in the excitation and inhibition network signaling has been implicated in multiple psychiatric disorders (*68*). Moreover, shifting the development of these networks by intervening during critical periods of development could have therapeutic potential (*69*, *70*). Previous studies have shown that systemic modulation of GABAergic neuron activity during an early critical period permanently normalizes excitation and inhibition balance in cortical regions such as the visual and insular cortex (*55*, *56*). Here, using the same diazepam treatment protocol, we could show that transient activation of inhibitory network function during an early critical period in ELA mice was able to reverse the observed social subordination behaviors. While the effects of diazepam were studied only in mice belonging to the ELA group, our findings do provide further support for the capacity of ELA to modulate the transcriptomic and physiological state of glutamatergic and GABAergic neurons of the vHPC in response to social defeat stress and its association with social hierarchies. However, future studies should further investigate the underlying mechanisms responsible for these effects. These studies should also embrace the use of complex and automated behavioral phenotyping systems, such as the SBs used in our study.

Overall, our findings have translational implications since adversity during early childhood can increase the likelihood of developing social anxiety disorders (*5*). Higher anxiety levels in turn have been linked to social dominance, where high anxiety individuals display lower competitive confidence (*71*). Our results suggest a potential therapeutic avenue where early intervention can permanently reverse some of the changes caused by ELA exposure in mice. Moreover, we provide evidence for the existence of cell type–specific vulnerabilities to ELA and significantly advance our understanding of how ELA exposure can affect the development of psychiatric disorders.

## MATERIALS AND METHODS

### Animals and housing

Male CD-1 (ICR) mice were group-housed in individually ventilated cages (IVCs) under specific pathogen–free conditions. The room was temperature-controlled (23 ± 1°C) and humidity-controlled (55 ± 10%) and on a 12-hour light-dark cycle (lights on at 7:00). Food and water were provided ad libitum. Animal experiments were approved by the Ethics Committee for the Care and Use of Laboratory Animals of the government of Upper Bavaria (Munich, Germany), or the Institutional Animal Care and Use Committee of the Weizmann Institute of Science (Rehovot, Israel).

### Limited bedding and nesting and acute social defeat stress

The limited bedding and nesting paradigm is a widely used and well-validated model for ELA (*33*). Breeding pairs, consisting of one male and one female adult CD-1 animal, were pair-housed. After 14 days, the male animal was removed. The day of birth was defined as PND 0. At PND 2, the litters were culled to a maximum of 10 pups and randomly assigned to the control or ELA condition. Control litters were placed in a standard IVC cage containing a standard amount of bedding material supplemented with two 5 cm × 5 cm cotton nestlets (Indulab, Gams, Switzerland). The ELA condition litters were placed in an IVC cage with reduced bedding material (50 ml), supplemented with half a cotton nestlet and a fine-gauge aluminum mesh (McNichols, Tampa, FL, USA) at the bottom. Both the control and the ELA dams and litters were left undisturbed until PND 9. At PND 9, all animals were returned to standard rearing conditions. For downstream experiments, mice were randomly assigned an experimental group (four mice per SB/cage). Each group consisted of two baseline (No ELA) and two ELA-exposed animals. For the acute social defeat stress, male resident CD-1 animals were trained for aggression. For three to five consecutive days, a naïve male CD-1 animal was placed in the home cage of the male resident CD-1 animal for approximately 5 min to induce aggressive attacks. Groups of animals were randomly assigned to the acute social defeat condition or the control condition. For the acute stress experiment (during adulthood), the entire group (two baseline and two ELA) was exposed to an individual social defeat session and placed back in their original group after the stress. This was done to prevent stress transfer from a stressed to an unstressed mouse, as shown by Sterley and colleagues (*72*). Furthermore, if a group was assigned to the acute defeat condition, then all members of that group were individually defeated at the same time. The animals to be acutely defeated were placed in the home cage of the resident aggressor animal, eliciting aggressive attacks toward the intruder animal. After the intruder animal displayed a full defeat posture (typically 1 to 2 min), it was placed back into the original group. Full defeat was characterized by an upright submissive position, limp forepaws, upwardly angled head, and retracted ears (*73*). On average, each mouse was defeated within the first 0.5 to 2 min of the interaction. This was dependent on the aggressiveness of the resident mouse. In addition, to avoid serious injuries, the subordinate mouse was separated by the experimenter immediately after adopting a defeat posture. To avoid any effects of acute stress caused by physically grabbing the mice out of their home cages or exposing them to a novel environment (without an aggressor), our control mice were left undisturbed in their home cages until tissue collection. The selection of mice for our sequencing experiments was based on two different criteria. First, we wanted three pairs for each comparison (Bl Ctrl versus ELA Ctrl and Bl Stress versus ELA Stress), where each pair originated from the same group (SB/cage). This was done to control and minimize for a batch effect produced by the group (SB/cage). Second, the selection for each animal pair was based on the results of our PCA analysis, with the constraint that they should show a high degree of separation between the ELA and baseline conditions (fig. S1, C and D). This resulted in three biological replicates from each of the four conditions, which were used for scRNA-seq analysis. For all experiments, animals were defeated at 8 a.m. and tissue was collected 5 hours after the acute social defeat stress.

### CORT measurement

CORT measurements were performed as previously described (*74*). Mouse trunk blood was taken and kept on ice until centrifugation (4°C). Plasma CORT levels were determined using radioimmunoassay according to the manufacturer’s protocol (MP Biomedicals, Eschwege, Germany). All samples were measured in duplicates.

### SB behavioral assessment

The behavior of mice was automatically tracked in the SB as described in detail previously (*35*, *38*, *75*). Groups of male nonsibling CD-1 mice consisting of two control and two ELA animals were housed together from weaning. The fur of the animals was painted different colors to keep track of their identity. Painting of the fur took less than 10 min and was performed under mild isoflurane anesthesia. The animals were allowed to recover before testing for at least several days. In the SB, food and water was provided ad libitum. This 60 cm × 60 cm living environment contained an enclosed nest and a smaller open nest, two feeders for water and food, an s-wall, and two ramps. During the light phase, the arenas were illuminated with around 200 lux and 2 lux during the dark phase (12 hours). Recording of the animals was done during the dark phase with a color-sensitive camera (Manta G-235C, Allied-Vision). The mouse tracking software was written in Matlab (MathWorks). The trajectory-based behavioral readouts, including the DS, are described in detail elsewhere (*35*). PCA analysis was performed in R using the mean values of all 58 readouts collected the first 2 days of SB tracking to assess the source of variance. The top two ranked PCs, namely, PC1 and PC2, containing the highest degree of variance were plotted. Significance of each of the individual 58 readouts was assessed in R using the “nlme” R package (*76*). Here, hierarchical linear mixed effects models were fit for each scaled and rank-normalized outcome. The condition and day were set as fixed effects, and the individual mice nested within groups were set as random effects. As mentioned above, only the first 2 days were considered in the analysis, as during this time period major differences across conditions have been shown to arise (*35*, *75*, *77*, *78*). This is likely due to the novelty and anxiety generated by the new environment, which exacerbated differences in exploration and prosocial and antagonistic interactions between the groups, which tend to normalize after this time point due to habituation to the environment.

### Tube test and *z* scoring

The tube test was performed as previously described (*40*). In brief, one ELA and one control animal were weaned in pairs. Animals were tested around 10 weeks of age. Animals were trained to go through a transparent 35-cm-long Plexiglas tube with an internal diameter of 34 mm for two consecutive days. Each training day, an animal went through the tube 10 times with alternating starting positions. The animal pairs were tested for three consecutive days, alternating the starting positions. Each training day consisted of three trails, where each pair was tested with alternating starting positions. The animal with the highest number of wins was considered the dominant animal. The testing environment was illuminated with 15 lux. *Z*-scores were calculated as described previously (*79*), integrating the total average number of wins, pushes, and percent time moving forward and retreating. The *z*-score of each of the individual behavior was calculated.

$$z=\frac{X-\mu }{\sigma }$$

These were combined in a total hierarchy score.

$$\text{Hierarchy score}=\frac{Z\mathrm{wins}+Z\mathrm{pushes}+Z\mathrm{forward}+Z\mathrm{retreat}}{\text{Number of tests}}$$

A higher score indicates an overall higher social rank.

### Single-cell suspensions, cell capture, and library preparation

Generation of single-cell suspensions and cell capture was performed as previously described (*50*). Five hours after acute social defeat stress, the mice received an isoflurane overdose and subsequently perfused with ice-cold phosphate-buffered saline (PBS). The brains were removed and placed in ice-cold oxygenated artificial cerebral spinal fluid (aCSF). The extracted tissue from each animal was kept separately and was maintained in the same ice-cold aCSF solution throughout the entire dissection and dissociation procedure. Throughout the experiment, the aCSF was oxygenated with a 5% CO_2_ in O_2_ mixture. Slices (1000 μm thick) were cut using a VT1200/S Leica vibratome. The vHPC (−2.46 mm bregma to −3.52 mm bregma) was manually extracted under guidance of a stereo microscope (M205C, Leica). The tissue was dissociated with the Papain dissociation system (Worthington) for 35 min at 37°C in a shaking water bath according to the provided protocol. Cells were filtered with 30-μm filters (Partec) and placed in cold aCSF. For sample loading, cell suspensions with ~1,000,000 cells/ml were used. Samples were loaded onto individual lanes of a 10x Genomics Chromium chip (10x Genomics), according to the manufacturer’s recommendations. Reverse transcription and library preparation were performed according to the protocol provided with the 10x Genomics Single-Cell v3.0 kit (10x Genomics). Library concentrations and fragment length were determined by qPCR using KAPA Library Quant (Kapa Biosystems) and Bioanalyzer High Sensitivity DNA kit (Agilent), respectively. The libraries were pooled and sequenced on a single lane of the Illumina NovaSeq 6000 System generating 100–base pair paired-end reads at a depth of ~330 million reads per sample.

### scRNA-seq preprocessing and QC

Cell Ranger software version 3.0.2 (10x Genomics) was used in the default mode to preprocess the data. To align and quantify the data, reference data for the mm10 assembly and gene annotation were provided by 10x Genomics. Following best practices guidelines (*47*), all further analyses were performed with Scanpy (version 1.4.5post3) (*80*). For QC assessment, the distribution of the number of genes, count depth, and the fraction of mitochondrial reads were determined. Since these were found to be fairly homogeneous, the same threshold was used for all samples. Cells with a count depth below 1000 and above 30,000, less than 500 genes, and a mitochondrial fraction above 0.2 were removed (for the QC plot, see fig. S5A). Additionally, genes expressed in less than 10 cells were removed as well. Doublet detection was performed using Scrublet (0.2.1) (*81*), with the following parameters: expected doublet rate, 2%; simulated doublet ratio, 3; number of neighbors, 15; minimum counts, 2; minimum cells, 3. The algorithm was run separately for each batch. This resulted in a total of 28,167 vHPC cells with 18,928 genes. For normalization, the size factors were determined separately on each sample using Scran (version 1.14.5) (*82*), and the input clusters were computed using the Louvain (0.6.1) (*48*) algorithm with a resolution of 0.2. This was followed by log+1 transformation of the data. Batch correction was done using Combat (*83*), available in Scanpy. The highly_variable_genes function selected the top 4000 highly variable genes. After PCA on the highly variable genes, we used the first 50 PCs for dimensionality reduction. Subsequently, for UMAP visualization (*49*), a *k*-nearest neighbor graph (KNN) graph (*k* = 15) was computed on the low-dimensional embedding.

### Single-cell clustering and annotation

Clustering was done using the Louvain (version 0.6.1) algorithm in Scanpy (*80*). We clustered at a resolution of *r* = 0.5. To identify marker genes for each cluster, the rank_genes_groups function in Scanpy was used on the log-normalized non–batch-corrected data, followed by a Welch’s *t* test comparing cells in the cluster with all other cells as reference. Cell type marker genes were identified from the literature and Gene Ontology for cell types using mousebrain.org (*50*, *51*, *84*). To further discriminate CA1, CA3, and DG neurons from one another, we used molecular markers for these populations in the vHPC that were provided by Cembrowski and colleagues (*52*). Using this information, we were able to further characterize various neuronal cell clusters by assigning them a specific anatomical location within the HPC.

### Differential expression analysis

Differential gene expression was calculated using the python package diffxpy (*53*). This analysis was performed pairwise comparing the four conditions in the following manner: baseline control versus baseline stress, baseline control versus ELA control, baseline stress versus ELA stress, and ELA control versus ELA stress. scRNA-seq data were modeled using a generalized linear model (GLM). In each cell cluster, we fit the following model:

$$Y_{ij}∼1+C_{j}+B_{j}$$

where *Y_ij_* is the log-normalized non–batch-corrected data of cell *j* and 1 is the intercept term. *C_j_* is the covariate that accounts for the condition (Bl Ctrl, Bl Stress, ELA Ctrl, or ELA Stress), and the technical covariate *B_j_* is used as a proxy for technical and biological factors that might influence gene expression. The test calculated *P* values for each gene, which were false discovery rate–corrected with the Benjamini-Hochberg method. Furthermore, we computed the mean expression of each gene within each of the four conditions. DEGs (*q*-value cutoff of 0.05) were filtered out if the mean expression was <0.5 in both conditions or if their log_2_FC was <|0.5|. Despite having a four-group setup, we chose to use a pairwise comparisons strategy. This is because, in this study, the conditions and batch effects were categorical and perfectly confounded such that there would be no sharing of information between covariates when fitting a joint model. Thus, fitting a pairwise model is computationally faster while giving the same results.

### Transcriptional patterning calculations

The DEGs identified in the four pairwise differential gene expression analyses (Bl Ctrl versus Bl Stress, Bl Ctrl versus ELA Ctrl, Bl Stress versus ELA Stress, and ELA Ctrl versus ELA Stress) were unified for each cell cluster into a single list of unique genes. The gene patterning score is based on the log_2_FC identified for each gene for each of the four comparisons

$$\mathrm{PatternScore}=|{\mathrm{FC}}_{a}-{\mathrm{FC}}_{d}|+(|{\mathrm{FC}}_{b}|+|{\mathrm{FC}}_{c}|)$$

where the absolute value of comparison FC_a_ = log_2_FC Bl Ctrl versus Bl Stress is subtracted by comparison FC_d_ = log_2_FC ELA Ctrl versus ELA Stress. This is subsequently modified by the sum of the absolute values of comparisons FC_b_ = log_2_FC Bl Ctrl versus ELA Ctrl and FC_c_ = log_2_FC Bl Stress versus ELA Stress. A higher gene patterning score indicates an increased likelihood for the presence of an interaction between ELA and acute stress.

Each of the distinct gene pattern scores was calculated using the mean expression values of each gene within each of the four conditions (Bl Ctrl, Bl Stress, ELA Ctrl, and ELA Stress), with the highest expression value set to one. Each of the gene patterns, namely, up- and down-regulated chronic, inverted, primed, and blunted, was calculated in the following manner:

ChronicUp=ΔBl−∣ΔELS∣−∣(BlStress−‖ELSStress,ELSCtrl‖)∣

ChronicDown=−ΔBl−∣ΔELS∣−∣(BlStress−‖ELSStress,ELSCtrl‖)∣

InvertedUp=−ΔBl+ΔELA−(∣BlCtrl−ELSCtrl∣)

InvertedDown=ΔBl−ΔELS−(∣BlCtrl−ELSCtrl∣)

PrimedUp=ΔELS−∣ΔBl∣−∣(ELSCtrl−‖BlCtrl,BlStress‖)∣

PrimedDown=−ΔELS−∣ΔBl∣−∣(ELSCtrl−‖BlCtrl,BlStress‖)∣

BluntedUp=ΔBl−∣ΔELS∣−∣(BlCtrl−‖ELSStress,ELSCtrl‖)∣

BluntedDown=−ΔBl−∣ΔELS∣−∣(BlCtrl−‖ELSStress,ELSCtrl‖)∣

where ΔBl = Bl Stress – Bl Ctrl and ΔELA = ELA Stress – ELA Ctrl.

### Metascape gene network analysis

The Metascape analysis was performed by dividing the DEGs from each cell cluster into three lists based on the gene patterning score, namely, the 25% percent highest and lowest scoring genes and the remaining 50% medium scoring genes. These three gene lists were uploaded using the “multiple gene list” setting and subsequently analyzed with the express analysis settings. This generated gene enrichment clusters based on the total set of DEGs with as overlay the contribution of the three (high, medium, and low) gene sets to each of the identified clusters. The *P* values associated with each of the gene enrichment clusters were hierarchical clustered and visualized as heatmaps using the Pheatmap package in R (*85*).

### RNAscope fluorescence in situ hybridization

Brains were removed and snap-frozen using 2-methylbutane and stored at −80°C until use. Frozen brains were cut with a cryostat in 20-μm-thick cryosections. RNAscope fluorescence in situ hybridization was performed according to the manufacturer’s instructions supplied with the RNAscope Fluorescent Multiplex Reagent Kit (Advanced Cell Diagnostics, Newark, CA, USA), as described previously (*86*). The following detection probes were used: *Rab3b* (Mm-Rab3b, catalog no. 501431), *Adarb2* (Mm-Adarb2-C3, catalog no. 519971-C3), *Nbl1* (Mm-Nbl1, catalog no. 454541), *Slc17a7* (Mm-Slc17a7-C2, catalog no. 416631-C2), and *Slc32a1* (Mm-Slc32a1-C2, catalog no. 319191-C2). Images were acquired with a Zeiss confocal microscope using a 20× objective using identical settings for all samples. The signal intensity of a target gene (*Rab3b*, *Adarb2*, and *Nbl1*) was measured in individual cells positive for the appropriated cell marker gene (*Slc17a7* or *Slc32a1*). Quantification of the images was done with Fiji. The mean signal intensity of all cells measured in an image field was averaged. For each condition, brain samples were collected from three animals. Two cryosections were selected and individually stained, each of which images was taken bilaterally.

### Ex vivo recordings

For brain slice preparation, mice were injected with pentobarbital (100 mg/kg, intraperitoneally) and perfused with carbogenated (95% O_2_, 5% CO_2_) ice-cold slicing solution containing 2.5 mM KCl, 11 mM glucose, 234 mM sucrose, 26 mM NaHCO_3_, 1.25 mM NaH_2_PO_4_, 10 mM MgSO_4_, 2 mM CaCl_2_; pH 7.4, 340 mOsm. After decapitation, 300-μm horizontal slices containing the vHPC were prepared in carbogenated ice-cold slicing solution using a vibratome (Leica VT 1200S) and allowed to recover for 20 min at 33°C in carbogenated high-osmolarity artificial cerebrospinal fluid (aCSF) containing 3.2 mM KCl, 11.8 mM glucose, 132 mM NaCl, 27.9 mM NaHCO_3_, 1.34 NaH_2_PO_4_, 1.07 mM MgCl_2_, 2.14 mM CaCl_2_; pH 7.4, 320 mOsm, followed by 40-min incubation at 33°C in carbogenated aCSF containing 3 mM KCl, 11 mM glucose, 123 mM NaCl, 26 mM NaHCO_3_, 1.25 mM NaH_2_PO_4_, 1 mM MgCl_2_, 2 mM CaCl_2_; pH 7.4, 300 mOsm. Subsequently, slices were kept at room temperature in carbogenated aCSF until use. CA1 pyramidal neurons were patched under visual guidance using infrared differential interference contrast (DIC) microscopy (BX51W1, Olympus) and an Andor Neo sCMOS camera (Oxford Instruments, Abingdon, UK). Borosilicate glass pipettes (BF100-58-10, Sutter Instrument, Novato, CA, USA) with resistances of 4 to 6 megohms were pulled using a laser micropipette puller (P-2000, Sutter Instrument) and filled with intracellular solution allowing EPSC and IPSC recording (120 mM cesium gluconate, 11 mM cesium chloride, 10 mM Hepes, 11 mM EGTA, 4 mM Mg-ATP, 0.3 mM Na_2_-GTP (guanosine triphosphate), 1 mM MgCl_2_, 1 mM CaCl_2_, 280 mM mOsm kg^−1^, pH adjusted to 7.3 with CsOH). All experiments were conducted at room temperature. In the recording chamber, slices were superfused with carbogenated aCSF (4 to 5 ml/min flow rate). Somatic whole-cell voltage-clamp recordings from CA1 pyramidal neurons (>1 gigohm seal resistance, −70 mV holding potential) were performed using a Multiclamp 700B amplifier (Molecular Devices, San Jose, CA, USA). Data were acquired using pClamp 10.7 on a personal computer connected to the amplifier via a Digidata-1440 interface (sampling rate: 20 kHz; low-pass filter: 8 kHz). Cells were clamped at either −70 mV or 0 mV to measure EPSCs and IPSCs, respectively. Data obtained with a series resistance of >20 megohms were discarded. Analysis was performed using ClampFit 10.7 and Easy Electrophysiology V2.2 (*87*).

### Diazepam treatment

The treatment regimen was performed as previously described (*55*). Briefly, ELA-exposed mice were randomly assigned to the control or diazepam treatment condition. Animals were intraperitoneally injected daily for 14 days with vehicle or diazepam (20 mg/kg in 0.9% saline) between PND 13 and 26.
