# Supplementary material for: Early life adversity shapes social subordination and cell type–specific transcriptomic patterning in the ventral hippocampus
Source: Sci Adv. 2023 Dec 1;9(48):eadj3793. doi: 10.1126/sciadv.adj3793 (PMC10691768; doi:10.1126/sciadv.adj3793)
Supplement: Supplementary file 1 — Figs. S1 to S12 Table S1 [file sciadv.adj3793_sm.pdf]

Science Advances
AAAS

Supplementary Materials for

Early life adversity shapes social subordination and cell type–specific transcriptomic patterning in the ventral hippocampus

Aron Kos *et al.*

Corresponding author: Alon Chen, alon.chen@weizmann.ac.il; Juan Pablo Lopez, jpablo.lopez@ki.se

Sci. Adv. **9**, eadj3793 (2023)
DOI: 10.1126/sciadv.adj3793

This PDF file includes:

Figs. S1 to S12
Table S1

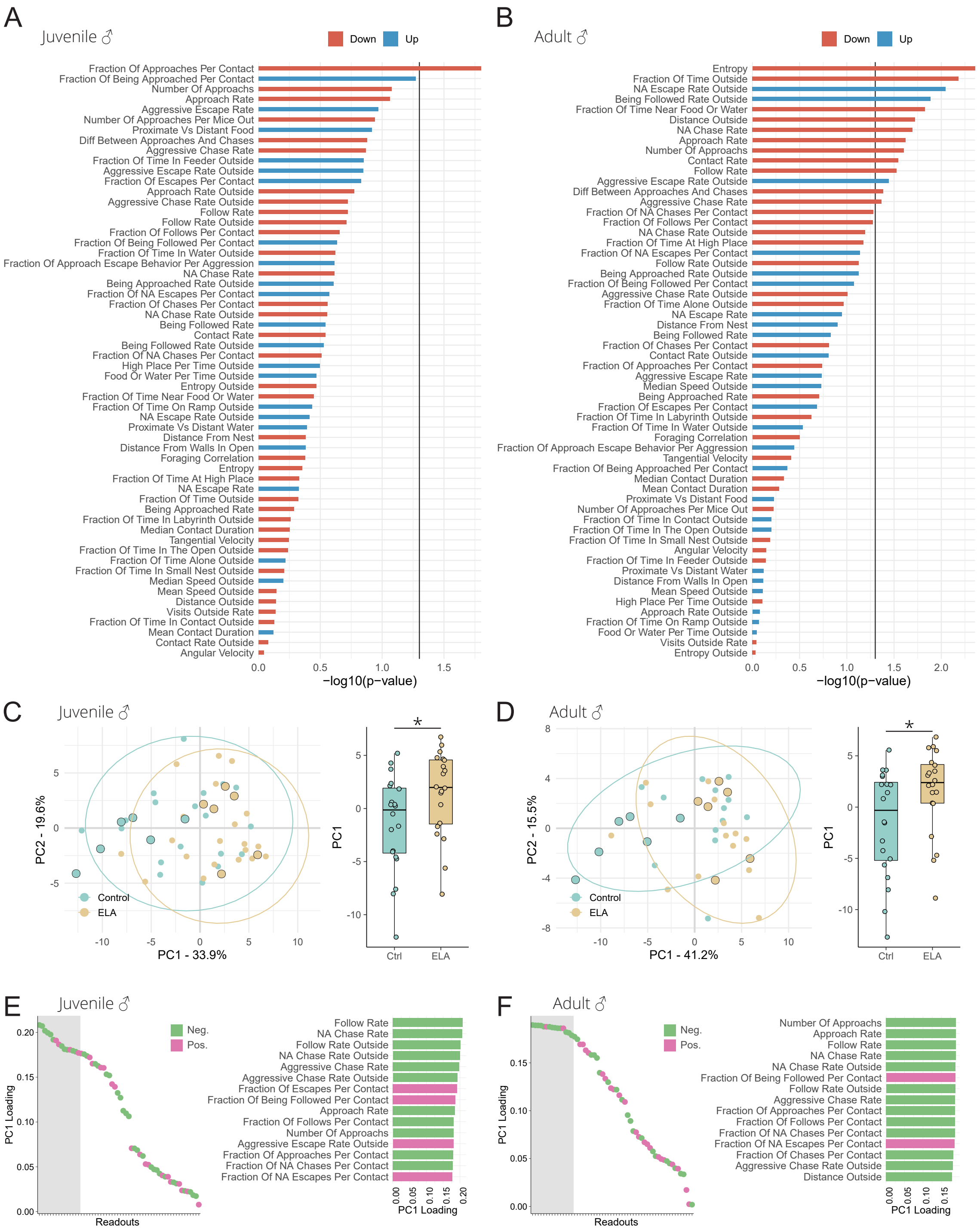

A
Juvenile ♂
Down
Up
Fraction Of Approaches Per Contact
Fraction Of Being Approached Per Contact
Number Of Approachs
Approach Rate
Aggressive Escape Rate
Number Of Approaches Per Mice Out
Proximate Vs Distant Food
Diff Between Approaches And Chases
Aggressive Chase Rate
Fraction Of Time In Feeder Outside
Aggressive Escape Rate Outside
Fraction Of Escapes Per Contact
Approach Rate Outside
Aggressive Chase Rate Outside
Follow Rate
Follow Rate Outside
Fraction Of Follows Per Contact
Fraction Of Being Followed Per Contact
Fraction Of Time In Water Outside
Fraction Of Approach Escape Behavior Per Aggression
NA Chase Rate
Being Approached Rate Outside
Fraction Of NA Escapes Per Contact
Fraction Of Chases Per Contact
NA Chase Rate Outside
Being Followed Rate
Contact Rate
Being Followed Rate Outside
Fraction Of NA Chases Per Contact
High Place Per Time Outside
Food Or Water Per Time Outside
Entropy Outside
Fraction Of Time Near Food Or Water
Fraction Of Time On Ramp Outside
NA Escape Rate Outside
Proximate Vs Distant Water
Distance From Nest
Distance From Walls In Open
Foraging Correlation
Entropy
Fraction Of Time At High Place
NA Escape Rate
Fraction Of Time Outside
Being Approached Rate
Fraction Of Time In Labyrinth Outside
Median Contact Duration
Tangential Velocity
Fraction Of Time In The Open Outside
Fraction Of Time Alone Outside
Fraction Of Time In Small Nest Outside
Median Speed Outside
Mean Speed Outside
Distance Outside
Visits Outside Rate
Fraction Of Time In Contact Outside
Mean Contact Duration
Contact Rate Outside
Angular Velocity
0.0
0.5
1.0
1.5
−log10(p−value)
B
Adult ♂
Down
Up
Entropy
Fraction Of Time Outside
NA Escape Rate Outside
Being Followed Rate Outside
Fraction Of Time Near Food Or Water
Distance Outside
NA Chase Rate
Approach Rate
Number Of Approachs
Contact Rate
Follow Rate
Aggressive Escape Rate Outside
Diff Between Approaches And Chases
Aggressive Chase Rate
Fraction Of NA Chases Per Contact
Fraction Of Follows Per Contact
NA Chase Rate Outside
Fraction Of Time At High Place
Fraction Of NA Escapes Per Contact
Follow Rate Outside
Being Approached Rate Outside
Fraction Of Being Followed Per Contact
Aggressive Chase Rate Outside
Fraction Of Time Alone Outside
NA Escape Rate
Distance From Nest
Being Followed Rate
Fraction Of Chases Per Contact
Contact Rate Outside
Fraction Of Approaches Per Contact
Aggressive Escape Rate
Median Speed Outside
Being Approached Rate
Fraction Of Escapes Per Contact
Fraction Of Time In Labyrinth Outside
Fraction Of Time In Water Outside
Foraging Correlation
Fraction Of Approach Escape Behavior Per Aggression
Tangential Velocity
Fraction Of Being Approached Per Contact
Median Contact Duration
Mean Contact Duration
Proximate Vs Distant Food
Number Of Approaches Per Mice Out
Fraction Of Time In Contact Outside
Fraction Of Time In The Open Outside
Fraction Of Time In Small Nest Outside
Angular Velocity
Fraction Of Time In Feeder Outside
Proximate Vs Distant Water
Distance From Walls In Open
Mean Speed Outside
High Place Per Time Outside
Approach Rate Outside
Fraction Of Time On Ramp Outside
Food Or Water Per Time Outside
Visits Outside Rate
Entropy Outside
0.0
0.5
1.0
1.5
2.0
−log10(p−value)
C
Juvenile ♂
PC2 - 19.6%
PC1 - 33.9%
Control
ELA
PC1
*
Ctrl
ELA
D
Adult ♂
PC2 - 15.5%
PC1 - 41.2%
Control
ELA
PC1
*
Ctrl
ELA
E
Juvenile ♂
Neg.
Pos.
PC1 Loading
Readouts
Follow Rate
NA Chase Rate
Follow Rate Outside
NA Chase Rate Outside
Aggressive Chase Rate
Aggressive Chase Rate Outside
Fraction Of Escapes Per Contact
Fraction Of Being Followed Per Contact
Approach Rate
Fraction Of Follows Per Contact
Number Of Approachs
Aggressive Escape Rate Outside
Fraction Of Approaches Per Contact
Fraction Of NA Chases Per Contact
Fraction Of NA Escapes Per Contact
PC1 Loading
F
Adult ♂
Neg.
Pos.
PC1 Loading
Readouts
Number Of Approachs
Approach Rate
Follow Rate
NA Chase Rate
NA Chase Rate Outside
Fraction Of Being Followed Per Contact
Follow Rate Outside
Aggressive Chase Rate
Fraction Of Approaches Per Contact
Fraction Of Follows Per Contact
Fraction Of NA Chases Per Contact
Fraction Of NA Escapes Per Contact
Fraction Of Chases Per Contact
Aggressive Chase Rate Outside
Distance Outside
PC1 Loading

Supplemental Figure 1: Social Box behavioral characterization of male early life adversity animals. (**A**-**B**) All 58 behavioral readouts collected from male mice over four days in the Social Box through automated location tracking at (**A**) juvenile and (**B**) adult stages. Red bars indicate an average decrease and blue bars indicate an average increase in readout values. Statistical differences were calculated between control and early life adversity (ELA) exposed mice. The black line indicates a significance threshold of -Log10 of 1.3. Hierarchical linear mixed effects models were fit for each rank-normalized and scaled outcome with condition and day as fixed effects and individual mice nested within groups as random effects. (**C-D**) Principal component analysis (PCA) using all 58 behavioral readouts with the top two PCs (PC1 and PC2) plotted for (**C**) juvenile and (**D**) adult animals. The percentages indicate the amount of variance explained for PC1 and PC2. The larger data points indicate the animals that were used for scRNA-seq analysis. The box plots (right) show a significant separation between control and ELA animals in PC1 both in (**C**) juvenile and (**D**) adult male mice. Unpaired *t* tests, two-tailed. (**E-F**) The top ranked contributing PC1 loadings, with the dot plot (left) visualizing the distributions of loadings for all 58 behaviors for (**E**) juvenile and (**F**) adult animals. The gray box insert indicates the top 15 selected highest loadings which are plotted on the right, with the negative loadings in green and positive loadings in pink. * $P < 0.05$.

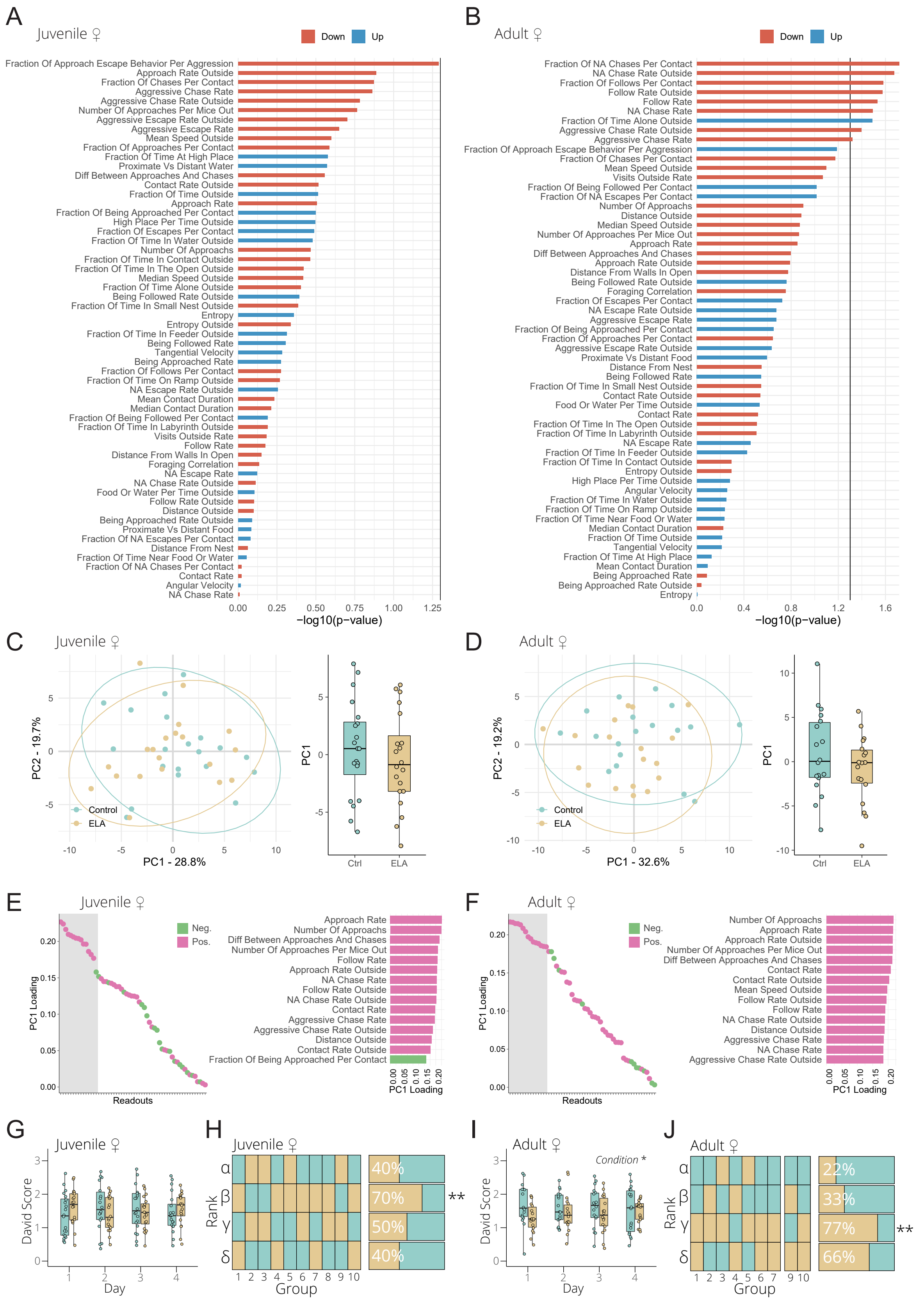
A
Juvenile ♀
Down
Up
Fraction Of Approach Escape Behavior Per Aggression
Approach Rate Outside
Fraction Of Chases Per Contact
Aggressive Chase Rate
Aggressive Chase Rate Outside
Number Of Approaches Per Mice Out
Aggressive Escape Rate Outside
Aggressive Escape Rate
Mean Speed Outside
Fraction Of Approaches Per Contact
Fraction Of Time At High Place
Proximate Vs Distant Water
Diff Between Approaches And Chases
Contact Rate Outside
Fraction Of Time Outside
Approach Rate
Fraction Of Being Approached Per Contact
High Place Per Time Outside
Fraction Of Escapes Per Contact
Fraction Of Time In Water Outside
Number Of Approachs
Fraction Of Time In Contact Outside
Fraction Of Time In The Open Outside
Median Speed Outside
Fraction Of Time Alone Outside
Being Followed Rate Outside
Fraction Of Time In Small Nest Outside
Entropy
Entropy Outside
Fraction Of Time In Feeder Outside
Being Followed Rate
Tangential Velocity
Being Approached Rate
Fraction Of Follows Per Contact
Fraction Of Time On Ramp Outside
NA Escape Rate Outside
Mean Contact Duration
Median Contact Duration
Fraction Of Being Followed Per Contact
Fraction Of Time In Labyrinth Outside
Visits Outside Rate
Follow Rate
Distance From Walls In Open
Foraging Correlation
NA Escape Rate
NA Chase Rate Outside
Food Or Water Per Time Outside
Follow Rate Outside
Distance Outside
Being Approached Rate Outside
Proximate Vs Distant Food
Fraction Of NA Escapes Per Contact
Distance From Nest
Fraction Of Time Near Food Or Water
Fraction Of NA Chases Per Contact
Contact Rate
Angular Velocity
NA Chase Rate
0.00 0.25 0.50 0.75 1.00 1.25
−log10(p−value)
B
Adult ♀
Down
Up
Fraction Of NA Chases Per Contact
NA Chase Rate Outside
Fraction Of Follows Per Contact
Follow Rate Outside
Follow Rate
NA Chase Rate
Fraction Of Time Alone Outside
Aggressive Chase Rate Outside
Aggressive Chase Rate
Fraction Of Approach Escape Behavior Per Aggression
Fraction Of Chases Per Contact
Mean Speed Outside
Visits Outside Rate
Fraction Of Being Followed Per Contact
Fraction Of NA Escapes Per Contact
Number Of Approachs
Distance Outside
Median Speed Outside
Number Of Approaches Per Mice Out
Approach Rate
Diff Between Approaches And Chases
Approach Rate Outside
Distance From Walls In Open
Being Followed Rate Outside
Foraging Correlation
Fraction Of Escapes Per Contact
NA Escape Rate Outside
Aggressive Escape Rate
Fraction Of Being Approached Per Contact
Fraction Of Approaches Per Contact
Aggressive Escape Rate Outside
Proximate Vs Distant Food
Distance From Nest
Being Followed Rate
Fraction Of Time In Small Nest Outside
Contact Rate Outside
Food Or Water Per Time Outside
Contact Rate
Fraction Of Time In The Open Outside
Fraction Of Time In Labyrinth Outside
NA Escape Rate
Fraction Of Time In Feeder Outside
Fraction Of Time In Contact Outside
Entropy Outside
High Place Per Time Outside
Angular Velocity
Fraction Of Time In Water Outside
Fraction Of Time On Ramp Outside
Fraction Of Time Near Food Or Water
Median Contact Duration
Fraction Of Time Outside
Tangential Velocity
Fraction Of Time At High Place
Mean Contact Duration
Being Approached Rate
Being Approached Rate Outside
Entropy
0.0 0.4 0.8 1.2 1.6
−log10(p−value)
C
Juvenile ♀
PC2 - 19.7%
PC1 - 28.8%
Control
ELA
PC1
Ctrl
ELA
D
Adult ♀
PC2 - 19.2%
PC1 - 32.6%
Control
ELA
PC1
Ctrl
ELA
E
Juvenile ♀
Neg.
Pos.
PC1 Loading
Readouts
Approach Rate
Number Of Approachs
Diff Between Approaches And Chases
Number Of Approaches Per Mice Out
Follow Rate
Approach Rate Outside
NA Chase Rate
Follow Rate Outside
NA Chase Rate Outside
Contact Rate
Aggressive Chase Rate
Aggressive Chase Rate Outside
Distance Outside
Contact Rate Outside
Fraction Of Being Approached Per Contact
PC1 Loading
F
Adult ♀
Neg.
Pos.
PC1 Loading
Readouts
Number Of Approachs
Approach Rate
Approach Rate Outside
Number Of Approaches Per Mice Out
Diff Between Approaches And Chases
Contact Rate
Contact Rate Outside
Mean Speed Outside
Follow Rate Outside
Follow Rate
NA Chase Rate Outside
Distance Outside
Aggressive Chase Rate
NA Chase Rate
Aggressive Chase Rate Outside
PC1 Loading
G
Juvenile ♀
David Score
Day
H
Juvenile ♀
Rank
α
β
γ
δ
Group
40%
70%
50%
40%
**
I
Adult ♀
Condition *
David Score
Day
J
Adult ♀
Rank
α
β
γ
δ
Group
22%
33%
77%
66%
**

Supplemental Figure 2: Social Box behavioral characterization of female early life adversity animals. (**A**-**B**) All 58 behavioral readouts collected from female mice over four days in the Social Box through automated location tracking at (**A**) juvenile and (**B**) adult stages. Red bars indicate an average decrease and blue bars indicate an average increase in readout values. Statistical differences were calculated between control and early life adversity (ELA) exposed mice. The black line indicates a significance threshold of -Log10 of 1.3. Hierarchical linear mixed effects models were fit for each rank-normalized and scaled outcome with condition and day as fixed effects and individual mice nested within groups as random effects. (**C-D**) Principal component analysis (PCA) using all 58 behavioral readouts with the top two PCs (PC1 and PC2) plotted for (**C**) juvenile and (**D**) adult animals. The percentages indicate the amount of variance explained for PC1 and PC2. The box plots (right) show no significant separation between control and ELA animals in PC1 for in (**C**) juvenile and (**D**) adult female mice. (**E-F**) The top ranked contributing PC1 loadings, with the dot plot (left) visualizing the distributions of loadings for all 58 behaviors for (**E**) juvenile and (**F**) adult animals. The gray box insert indicates the top 15 selected highest loadings which are plotted on the right, with the negative loadings in green and positive loadings in pink. ELA exposed animals have a lower daily calculated David score based on chases at the (**G**) juvenile and (**I**) adult stage. Two-way ANOVA repeated measures. (**H, J**) The hierarchy distribution based on the cumulative David score over four days of all ten groups tested in the SB. The hierarchy order is from alpha, beta, gamma to sigma, with the highest-ranking animal being the alpha. Both at the (**H**) juvenile and (**J**) adult stage, only adult female ELA mice display a significantly lower social rank. Group eight was removed due to death of one of the group members. Yates' corrected Chi-square test. * P < 0.05, ** P < 0.01.

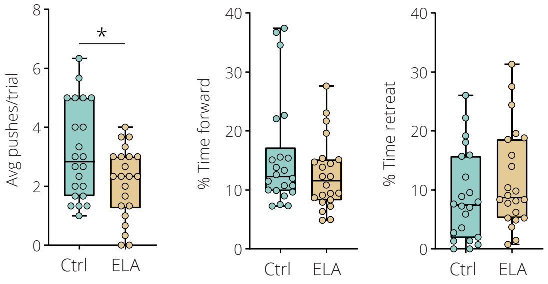

Avg pushes/trial
% Time forward
% Time retreat
*
0
2
4
6
8
0
10
20
30
40
Ctrl
ELA

Supplemental Figure 3: Tube test behaviors of early life adversity animals. Quantification of (**A**) the average number of pushes, (**B**) percentage of total time moving forward and (**C**) percentage of total time retreating in the tube test. Early life adversity (ELA) exposed animals push significantly less compared to control animals. Box plots represent the 25%, median and 75% quartile, whiskers span minimum to maximum. Unpaired *t* tests, two-tailed. * P < 0.05.

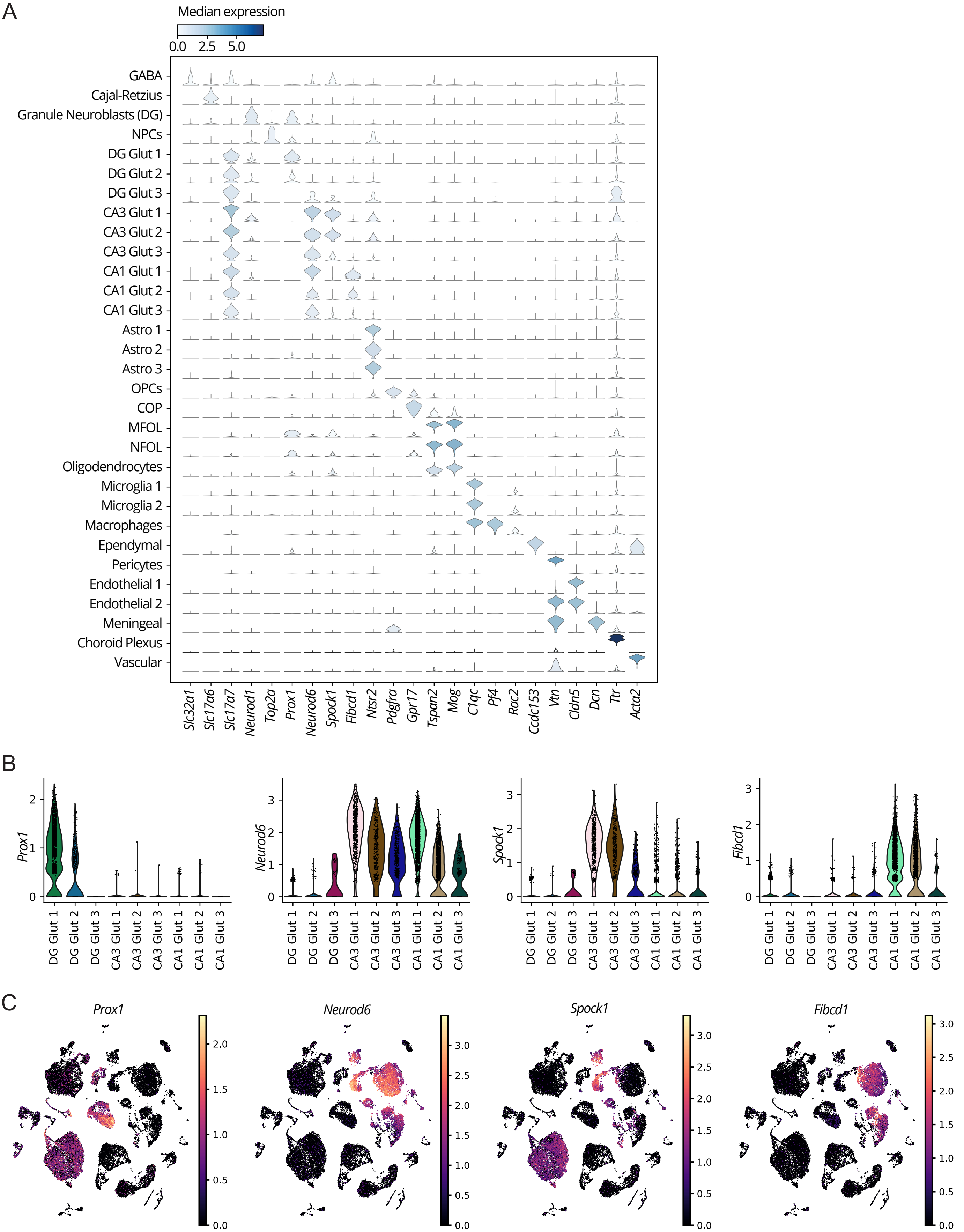

A
Median expression
0.0
2.5
5.0
GABA
Cajal-Retzius
Granule Neuroblasts (DG)
NPCs
DG Glut 1
DG Glut 2
DG Glut 3
CA3 Glut 1
CA3 Glut 2
CA3 Glut 3
CA1 Glut 1
CA1 Glut 2
CA1 Glut 3
Astro 1
Astro 2
Astro 3
OPCs
COP
MFOL
NFOL
Oligodendrocytes
Microglia 1
Microglia 2
Macrophages
Ependymal
Pericytes
Endothelial 1
Endothelial 2
Meningeal
Choroid Plexus
Vascular
Slc32a1
Slc17a6
Slc17a7
Neurod1
Top2a
Prox1
Neurod6
Spock1
Fibcd1
Ntsr2
Pdgfra
Gpr17
Tspan2
Mog
C1qc
Pf4
Rac2
Ccdc153
Vtn
Cldn5
Dcn
Ttr
Acta2
B
Prox1
Neurod6
Spock1
Fibcd1
DG Glut 1
DG Glut 2
DG Glut 3
CA3 Glut 1
CA3 Glut 2
CA3 Glut 3
CA1 Glut 1
CA1 Glut 2
CA1 Glut 3
C
Prox1
Neurod6
Spock1
Fibcd1

Supplemental Figure 4: Identity of cell clusters in the ventral hippocampus and marker genes. (**A**) Staggered violin plot of expression patterns of cell-type-specific identity markers. (**B**) Violin plots showing the expression of *Prox1*, *Neurod6*, *Spock1* and *Fibcd1* across the neuronal cell clusters. (**C**) Uniform Manifold Approximation and Projection (UMAP) plots in which the color gradient indicates the degree of expression of *Prox1*, *Neurod6*, *Spock1* and *Fibcd1* across all cell clusters.

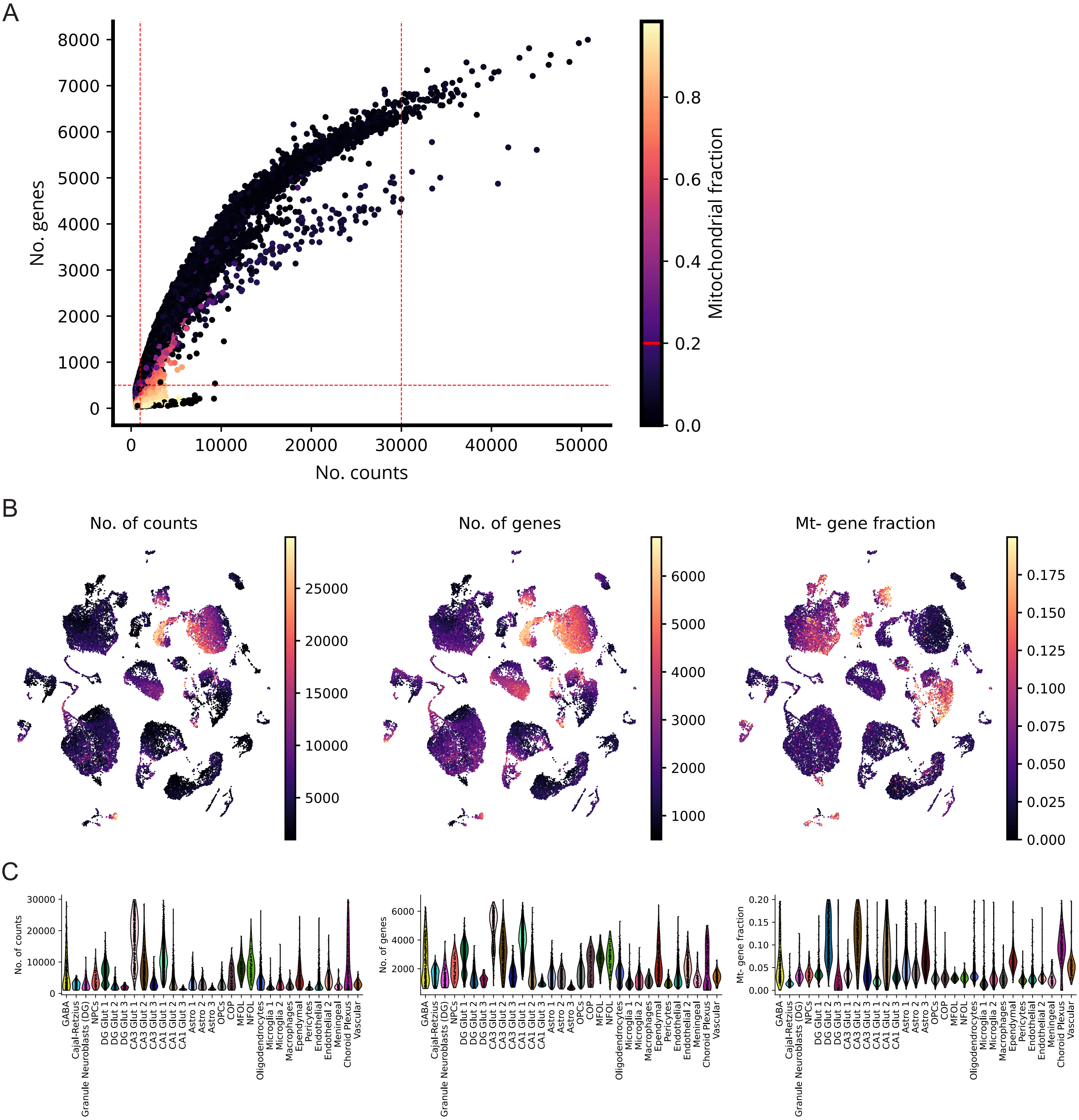
A
No. genes
No. counts
Mitochondrial fraction
8000
7000
6000
5000
4000
3000
2000
1000
0
10000
20000
30000
40000
50000
0.8
0.6
0.4
0.2
0.0
B
No. of counts
No. of genes
Mt- gene fraction
25000
20000
15000
10000
5000
6000
5000
4000
3000
2000
1000
0.175
0.150
0.125
0.100
0.075
0.050
0.025
0.000
C
No. of counts
No. of genes
Mt- gene fraction
GABA
Cajal-Retzius
Granule Neuroblasts (DG)
NPCs
DG Glut 1
DG Glut 2
DG Glut 3
CA3 Glut 1
CA3 Glut 2
CA3 Glut 3
CA1 Glut 1
CA1 Glut 2
CA1 Glut 3
Astro 1
Astro 2
Astro 3
OPCs
COP
MFOL
NFOL
Oligodendrocytes
Microglia 1
Microglia 2
Macrophages
Ependymal
Pericytes
Endothelial
Endothelial 2
Meningeal
Choroid Plexus
Vascular

Supplemental Figure 5: Quality control of scRNA-seq data. (**A**) Scatterplot of the count depth and number of genes expressed in each cell, color gradient indicates the fraction of counts of mitochondrial genes. The red dashed lines indicate the QC thresholds that were used. Cells with a count depth below a 1000 and above 30000, and less than 500 genes were removed. Furthermore, cells with a mitochondrial fraction above 0.2 were removed. (**B**) UMAP projections of all cell clusters where the color gradient indicates the number of counts, genes, and mitochondrial enrichment respectively. (**C**) Violin plots of the number of counts, genes, and mitochondrial enrichment per identified cell cluster.

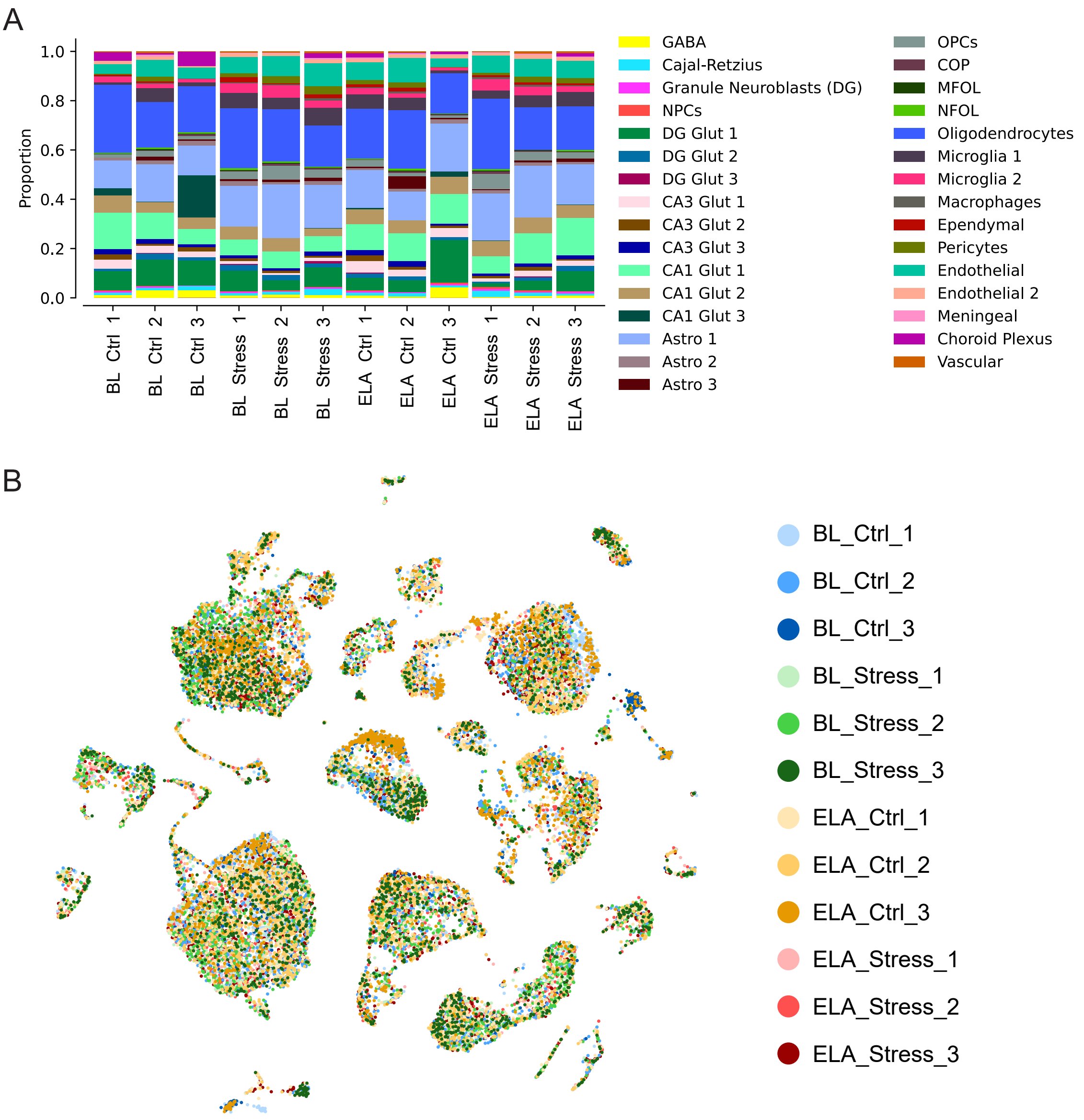

A
Proportion
0.0
0.2
0.4
0.6
0.8
1.0
BL Ctrl 1
BL Ctrl 2
BL Ctrl 3
BL Stress 1
BL Stress 2
BL Stress 3
ELA Ctrl 1
ELA Ctrl 2
ELA Ctrl 3
ELA Stress 1
ELA Stress 2
ELA Stress 3
GABA
Cajal-Retzius
Granule Neuroblasts (DG)
NPCs
DG Glut 1
DG Glut 2
DG Glut 3
CA3 Glut 1
CA3 Glut 2
CA3 Glut 3
CA1 Glut 1
CA1 Glut 2
CA1 Glut 3
Astro 1
Astro 2
Astro 3
OPCs
COP
MFOL
NFOL
Oligodendrocytes
Microglia 1
Microglia 2
Macrophages
Ependymal
Pericytes
Endothelial
Endothelial 2
Meningeal
Choroid Plexus
Vascular
B
BL_Ctrl_1
BL_Ctrl_2
BL_Ctrl_3
BL_Stress_1
BL_Stress_2
BL_Stress_3
ELA_Ctrl_1
ELA_Ctrl_2
ELA_Ctrl_3
ELA_Stress_1
ELA_Stress_2
ELA_Stress_3

Supplemental Figure 6: Cell-type proportions and distribution across biological replicates. (**A**) Stacked bar plot showing the proportion of each cell type per biological replicate from all four conditions. (**B**) UMAP plot showing the distribution of cells for each biological replicate from all four conditions.

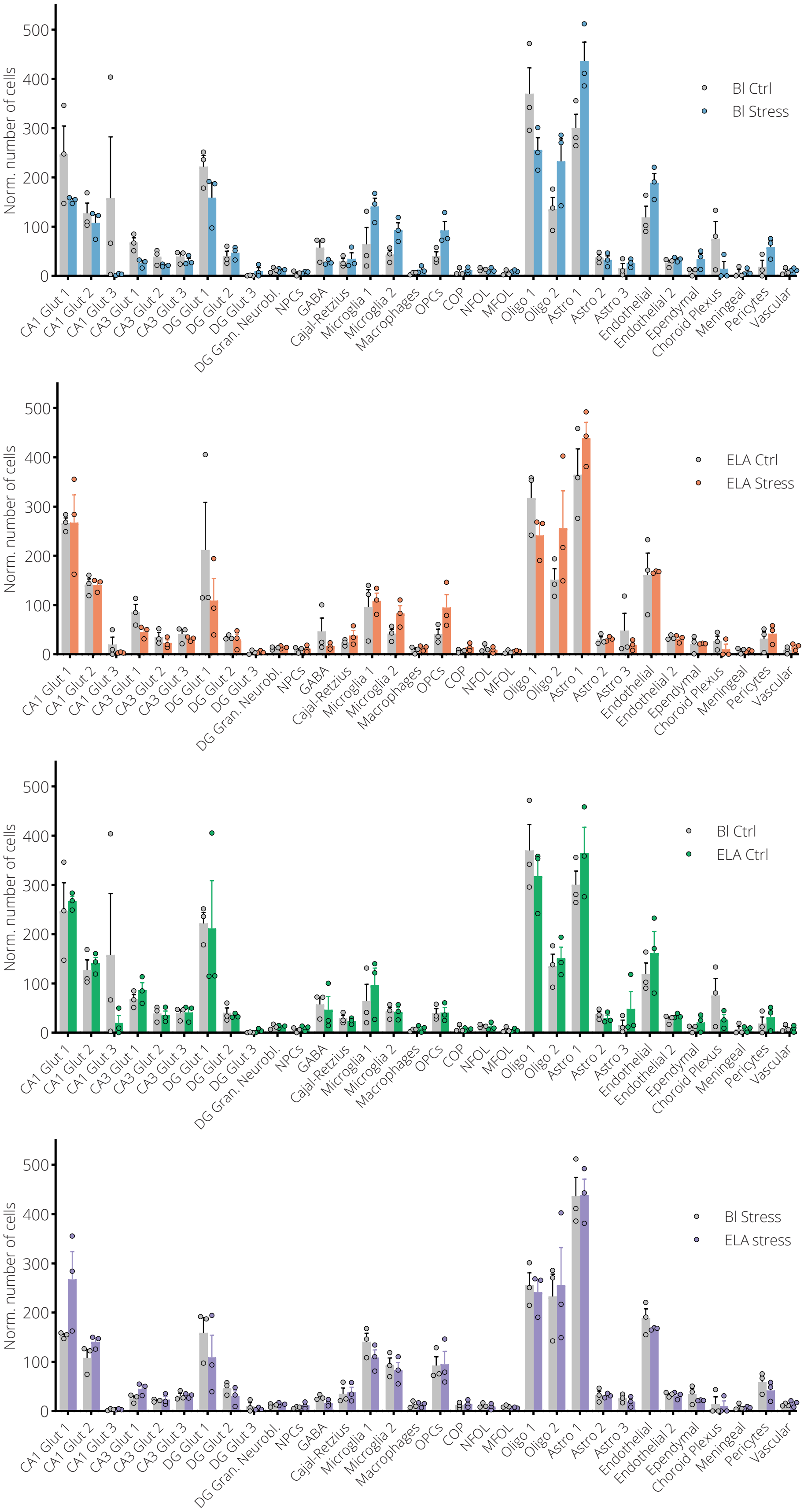
Norm. number of cells
0
100
200
300
400
500
Bl Ctrl
Bl Stress
ELA Ctrl
ELA Stress
Bl Ctrl
ELA Ctrl
Bl Stress
ELA stress
CA1 Glut 1
CA1 Glut 2
CA1 Glut 3
CA3 Glut 1
CA3 Glut 2
CA3 Glut 3
DG Glut 1
DG Glut 2
DG Glut 3
DG Gran. Neurobl.
NPCs
GABA
Cajal-Retzius
Microglia 1
Microglia 2
Macrophages
OPCs
COP
NFOL
MFOL
Oligo 1
Oligo 2
Astro 1
Astro 2
Astro 3
Endothelial
Endothelial 2
Ependymal
Choroid Plexus
Meningeal
Pericytes
Vascular

Supplemental Figure 7: Distribution in cell numbers per cluster between conditions. No significant differences were identified in the number of cells between cell clusters between the four comparisons (**A**) baseline control (Bl Ctrl), (**B**) baseline stress (Bl Stress), (**C**) early life stress control (ELS Ctrl) and (**D**) ELS stress.

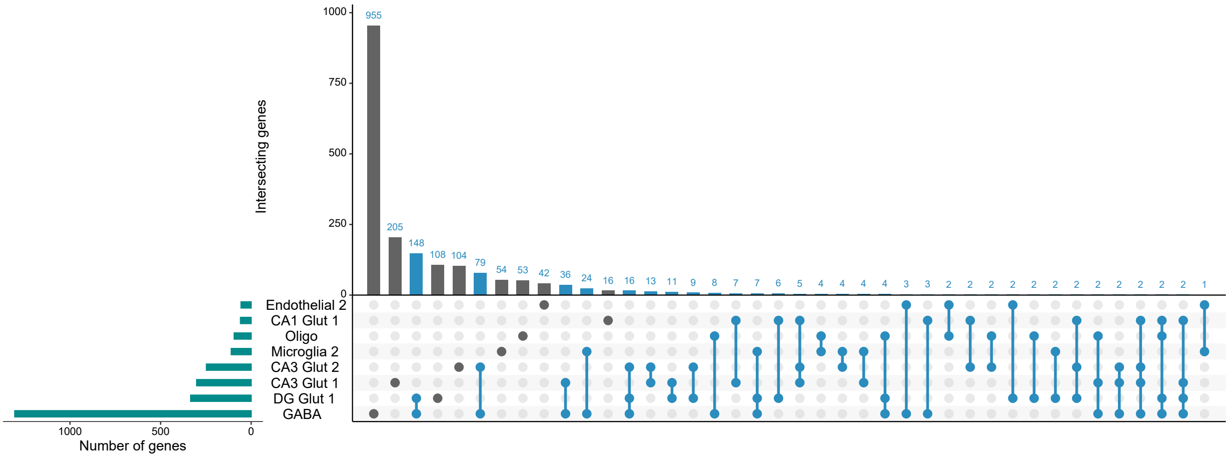

Intersecting genes
1000
750
500
250
0
955
205
148
108
104
79
54
53
42
36
24
16
16
13
11
9
8
7
7
6
5
4
4
4
4
3
3
2
2
2
2
2
2
2
2
2
2
2
2
1
Endothelial 2
CA1 Glut 1
Oligo
Microglia 2
CA3 Glut 2
CA3 Glut 1
DG Glut 1
GABA
1000
500
0
Number of genes

Supplemental Figure 8: Unique or overlapping differential expressed genes between cell clusters. UpSet plot showing unique and overlapping genes. The top eight clusters with the highest number of differentially expressed genes (DEGs) are shown. The left bar plot (dark green) indicates the number of DEGs in each cluster. Most DEGs are unique to each cluster indicated with the dark grey dots (below), the dark grey bars on top indicate the number of genes. The dark blue lines intersecting with the dark blue dots in the lower section indicate overlapping gene sets between clusters. The blue bars on top indicate the number of unique overlapping genes for each of the intersections.

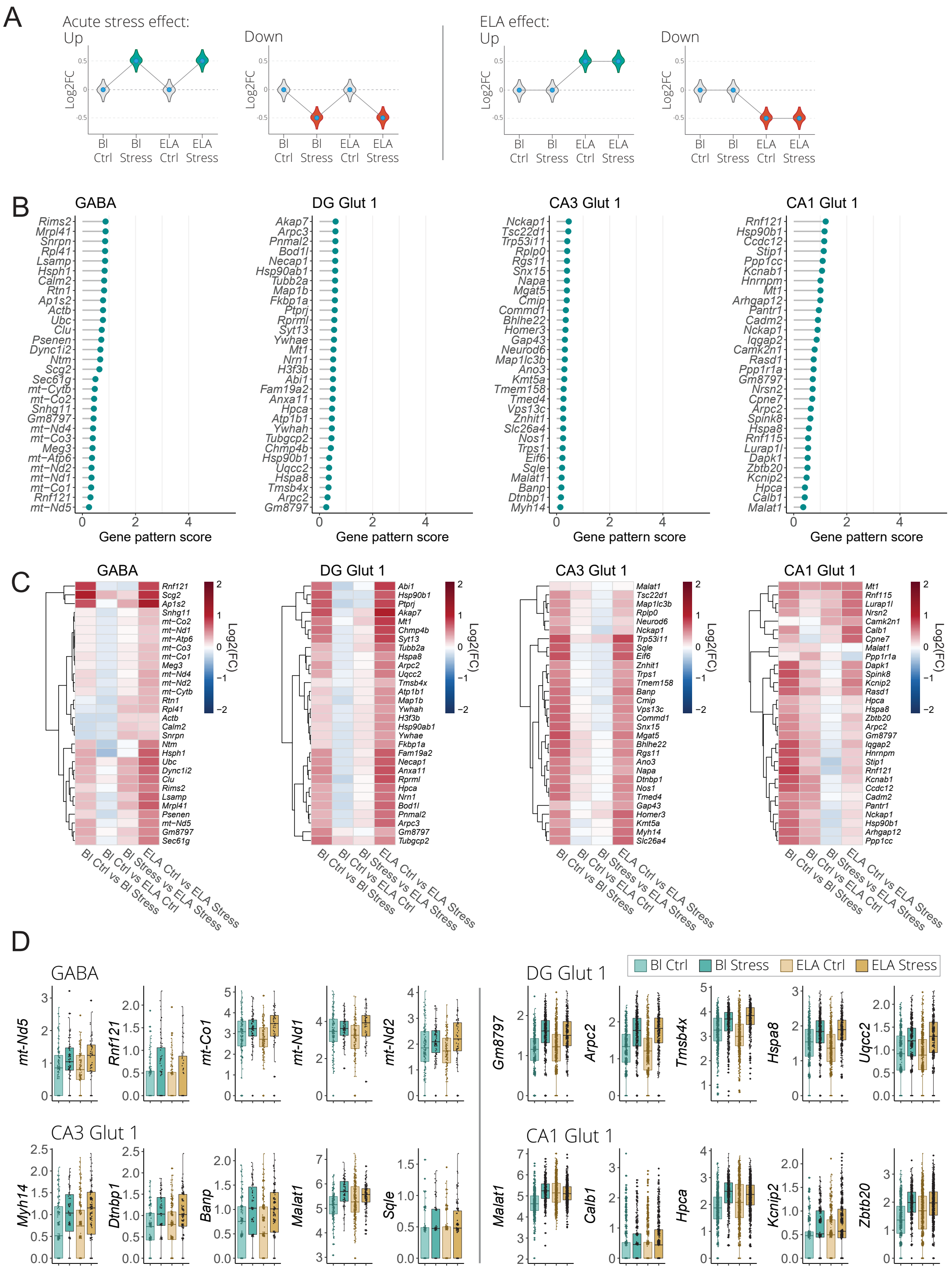

A
Acute stress effect:
Up
Down
ELA effect:
Up
Down
Log2FC
Bl Ctrl
Bl Stress
ELA Ctrl
ELA Stress
B
GABA
DG Glut 1
CA3 Glut 1
CA1 Glut 1
Gene pattern score
C
GABA
DG Glut 1
CA3 Glut 1
CA1 Glut 1
Log2(FC)
Bl Ctrl vs Bl Stress
Bl Ctrl vs ELA Ctrl
Bl Stress vs ELA Stress
ELA Ctrl vs ELA Stress
D
GABA
DG Glut 1
CA3 Glut 1
CA1 Glut 1
Bl Ctrl
Bl Stress
ELA Ctrl
ELA Stress
mt-Nd5
Rnf121
mt-Co1
mt-Nd1
mt-Nd2
Gm8797
Arpc2
Tmsb4x
Hspa8
Uqcc2
Myh14
Dtnbp1
Banp
Malat1
Sqle
Malat1
Calb1
Hpca
Kcnip2
Zbtb20

Supplemental Figure 9: Least transcriptionally patterned differential expressed genes. (**A**) Illustration of the theoretical expression changes induced by acute stress and early life adversity (ELA). With these expression patterns there is no interaction between the two. Genes with a low gene pattern score have a higher likelihood of showing one of the theoretical expression patterns. (**B**) Top 30 ranked lowest scoring genes in the GABAergic neurons (GABA), dentate gyrus glutamatergic neurons cluster 1 (DG Glut 1), CA3 glutamatergic neurons cluster 1 (CA3 Glut 1) and CA1 glutamatergic neurons cluster 1 (CA1 Glut 1). (**C**) Clustered heatmap visualization of the Log2FC of the top 30 lowest scoring genes in the four selected clusters. The four rows in each heatmap the four differential expression comparisons. (**D**) Expression patterns quantified by scRNA-seq of the five lowest patterned genes shows that the lowest scoring genes are primarily acute stress up regulated genes which are unaffected by the ELA background. Box plots represent the 25%, median and 75% quartile, whiskers span 1.5 x IQR.

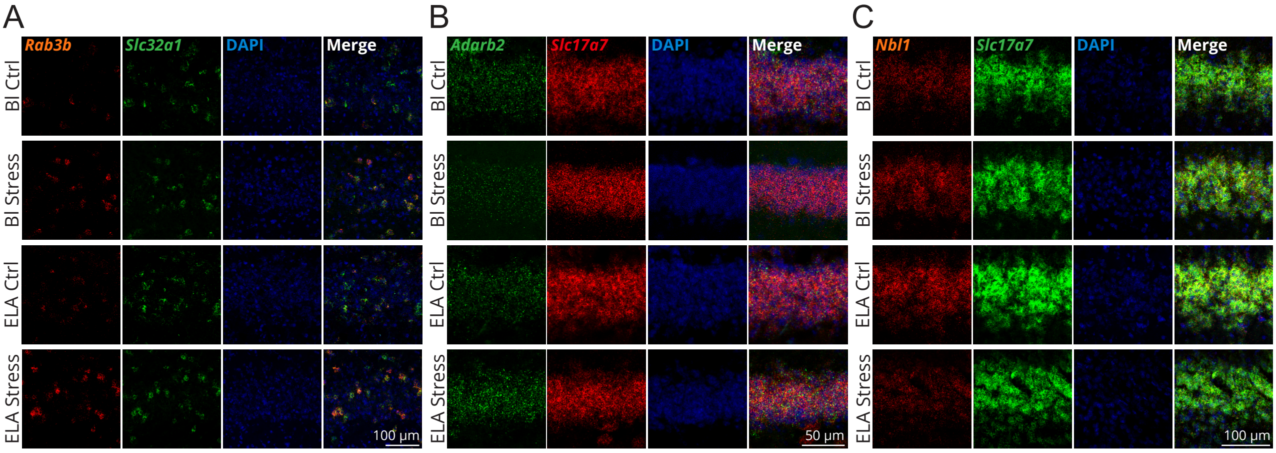
A
Rab3b
Slc32a1
DAPI
Merge
Bl Ctrl
Bl Stress
ELA Ctrl
ELA Stress
100 µm
B
Adarb2
Slc17a7
DAPI
Merge
Bl Ctrl
Bl Stress
ELA Ctrl
ELA Stress
50 µm
C
Nbl1
Slc17a7
DAPI
Merge
Bl Ctrl
Bl Stress
ELA Ctrl
ELA Stress
100 µm

Supplemental Figure 10. *Rab3b*, *Adarb2* and *Nbl1* differentially respond to acute stress after early life adversity exposure. (A-C) Representative images of RNAscope fluorescence *in situ* hybridization-stained ventral hippocampus (vHPC) slices collected from the baseline control (Bl Ctrl), baseline stress (Bl Stress), early life adversity (ELA) control (ELA Ctrl) and ELA stress conditions.

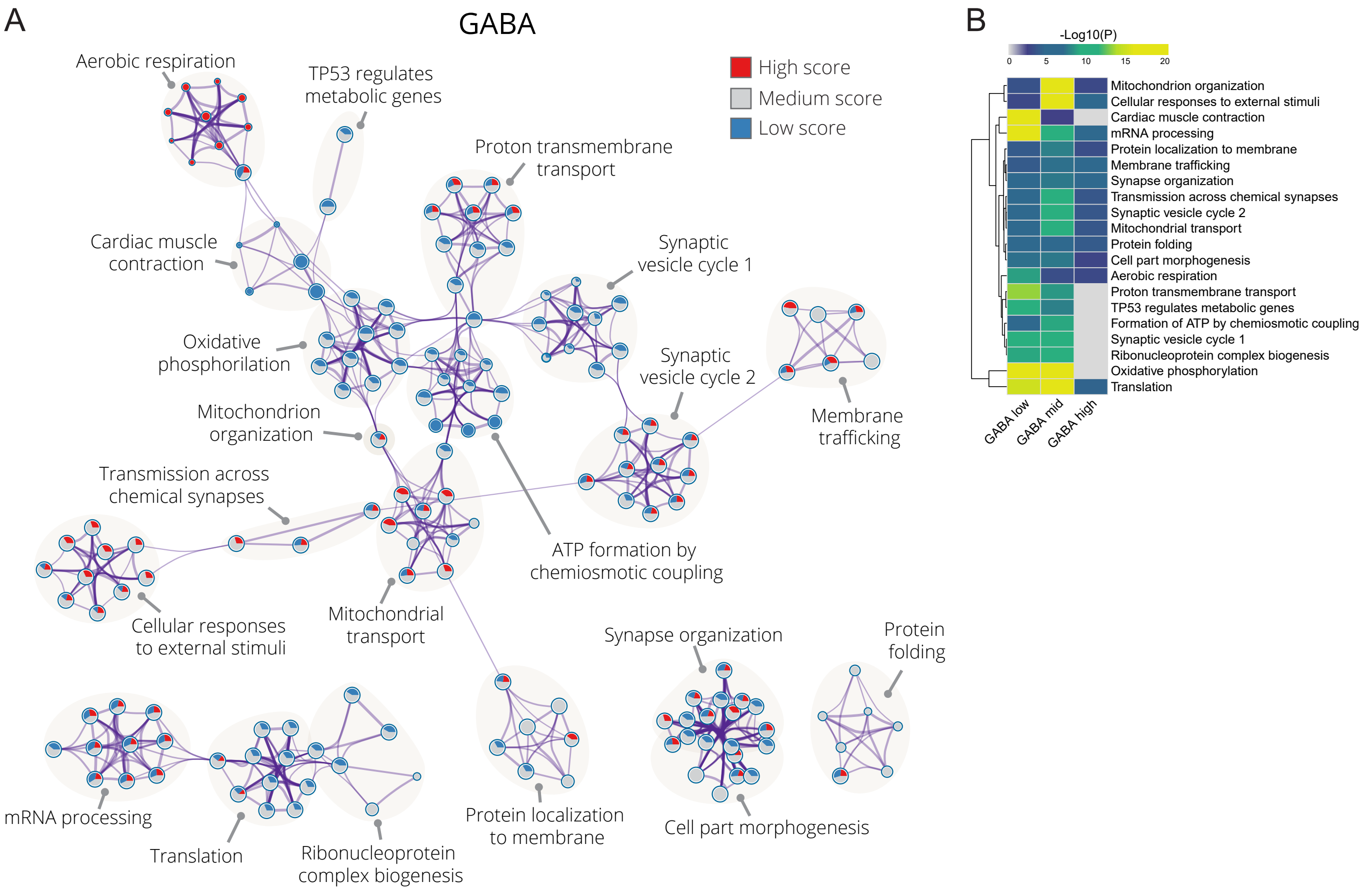
A
GABA
High score
Medium score
Low score
Aerobic respiration
TP53 regulates metabolic genes
Proton transmembrane transport
Cardiac muscle contraction
Synaptic vesicle cycle 1
Oxidative phosphorilation
Synaptic vesicle cycle 2
Mitochondrion organization
Membrane trafficking
Transmission across chemical synapses
ATP formation by chemiosmotic coupling
Cellular responses to external stimuli
Mitochondrial transport
Synapse organization
Protein folding
mRNA processing
Translation
Ribonucleoprotein complex biogenesis
Protein localization to membrane
Cell part morphogenesis
B
-Log10(P)
0
5
10
15
20
Mitochondrion organization
Cellular responses to external stimuli
Cardiac muscle contraction
mRNA processing
Protein localization to membrane
Membrane trafficking
Synapse organization
Transmission across chemical synapses
Synaptic vesicle cycle 2
Mitochondrial transport
Protein folding
Cell part morphogenesis
Aerobic respiration
Proton transmembrane transport
TP53 regulates metabolic genes
Formation of ATP by chemiosmotic coupling
Synaptic vesicle cycle 1
Ribonucleoprotein complex biogenesis
Oxidative phosphorylation
Translation
GABA low
GABA mid
GABA high

Supplemental Figure 11: Early life adversity susceptible or resilient differentially expressed gene networks in GABAergic neurons. (**A**) Metascape enrichment network visualization of the differentially expressed genes (DEGs) within the GABAergic neuron cluster identifying gene clusters more-or-less susceptible for early life adversity (ELA) induced gene patterning. The color code within each node represents the top 25% highest gene patterning scoring genes (red), 25% lowest scoring genes (blue) and the remaining 50% medium scoring genes (grey). (**B**) Clustered heatmap visualization of the -Log10 p-value associated with each low, medium and high scoring gene pathway.

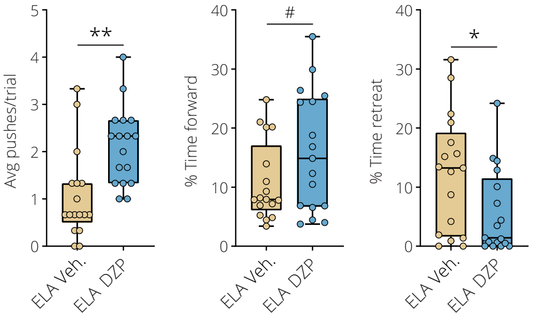

5
4
3
2
1
0
Avg pushes/trial
**
ELA Veh.
ELA DZP
40
30
20
10
0
% Time forward
#
ELA Veh.
ELA DZP
40
30
20
10
0
% Time retreat
*
ELA Veh.
ELA DZP

Supplemental Figure 12: Tube test behaviors of diazepam treated early life adversity animals. Quantification of the average number of pushes, percentage of total time moving forward and percentage of total time retreating in the tube test. Early life adversity (ELA) exposed animals chronically treated with diazepam (DZP) (**A**) push significantly more, (**B**) spend less time moving forward and (**C**) retreat less compared to saline (Sal.) injected ELA animals. Box plots represent the 25%, median and 75% quartile, whiskers span minimum to maximum. Unpaired *t* tests, two-tailed. # P < 0.1, * P < 0.05, ** P < 0.01.

Condition comparison	Cluster	Gene	qval	Log2fc	Mean expression condition 1	Mean expression condition 2
Bl Ctrl Bl Stress	GABA	*Rab3b*	0.961329062	-0.09928088	0.329000295	0.315773255
Bl Ctrl ELA Ctrl	GABA	*Rab3b*	0.198631654	-0.43176524	0.329000295	0.237828506
Bl Stress ELA Stress	GABA	*Rab3b*	0.013448836	1.06230425	0.315773255	0.716044298
ELA Ctrl ELA Stress	GABA	*Rab3b*	1.04253E-05	1.396774697	0.237828506	0.716044298
Bl Ctrl Bl Stress	DG Glut 1	*Adarb2*	2.01196E-11	-0.59244313	0.464311451	0.296669075
Bl Ctrl Bl Stress	DG Glut 2	*Adarb2*	0.755600275	-0.46406836	0.414567779	0.254720346
Bl Ctrl ELA Ctrl	DG Glut 1	*Adarb2*	0.000520172	-0.35336791	0.464311451	0.309501342
Bl Ctrl ELA Ctrl	DG Glut 2	*Adarb2*	0.92554625	0.143991672	0.414567779	0.421222124
Bl Stress ELA Stress	DG Glut 1	*Adarb2*	1.00E-16	1.094626153	0.296669075	0.714702507
Bl Stress ELA Stress	DG Glut 2	*Adarb2*	0.013611405	0.827408617	0.254720346	0.565356487
ELA Ctrl ELA Stress	DG Glut 1	*Adarb2*	1.00E-16	0.862763853	0.309501342	0.714702507
ELA Ctrl ELA Stress	DG Glut 2	*Adarb2*	0.762748614	0.219604158	0.421222124	0.565356487
Bl Ctrl Bl Stress	NPCs	*Nbl1*	0.394199516	0.791705092	0.389057364	0.715483177
Bl Ctrl Bl Stress	CA3 Glut 1	*Nbl1*	1.00E-16	0.967374641	0.6749151	1.317506979
Bl Ctrl Bl Stress	CA3 Glut 2	*Nbl1*	0.006388961	0.803418092	0.361598856	0.72580065
Bl Ctrl Bl Stress	CA3 Glut 3	*Nbl1*	0.014754223	0.933468524	0.206134232	0.469054445
Bl Ctrl Bl Stress	CA1 Glut 1	*Nbl1*	1.00E-16	0.983139202	0.254165402	0.615394128
Bl Ctrl Bl Stress	Pericytes	*Nbl1*	0.838437814	-0.18608254	1.403872086	1.226873885
Bl Ctrl Bl Stress	Endothelial 2	*Nbl1*	0.759321579	0.122255658	0.926700766	1.087642454
Bl Ctrl Bl Stress	Vascular	*Nbl1*	0.933591124	-0.15396678	0.793638067	0.762037313
Bl Ctrl ELA Ctrl	NPCs	*Nbl1*	0.972646739	-0.09616122	0.389057364	0.39914922
Bl Ctrl ELA Ctrl	CA3 Glut 1	*Nbl1*	1.00E-16	0.776730683	0.6749151	1.092528095
Bl Ctrl ELA Ctrl	CA3 Glut 2	*Nbl1*	3.08733E-05	0.952259865	0.361598856	0.778015737
Bl Ctrl ELA Ctrl	CA3 Glut 3	*Nbl1*	4.47922E-11	1.463265272	0.206134232	0.734353096
Bl Ctrl ELA Ctrl	CA1 Glut 1	*Nbl1*	1.00E-16	1.094316306	0.254165402	0.619767306
Bl Ctrl ELA Ctrl	Pericytes	*Nbl1*	0.901086896	-0.30447157	1.403872086	1.139461906
Bl Ctrl ELA Ctrl	Endothelial 2	*Nbl1*	0.952999207	0.074124773	0.926700766	0.912775269
Bl Ctrl ELA Ctrl	Vascular	*Nbl1*	0.895257603	-0.22261114	0.793638067	0.678607903
Bl Stress ELA Stress	NPCs	*Nbl1*	0.319827467	-0.81921787	0.715483177	0.392016392
Bl Stress ELA Stress	CA3 Glut 1	*Nbl1*	1.00E-16	-0.86316894	1.317506979	0.699924851
Bl Stress ELA Stress	CA3 Glut 2	*Nbl1*	0.008471502	-0.96803819	0.72580065	0.321384128
Bl Stress ELA Stress	CA3 Glut 3	*Nbl1*	0.288385557	-0.66750212	0.469054445	0.25781934
Bl Stress ELA Stress	CA1 Glut 1	*Nbl1*	1.00E-16	-0.77894523	0.615394128	0.301681546
Bl Stress ELA Stress	Pericytes	*Nbl1*	0.705578511	-0.12347337	1.226873885	1.173891677
Bl Stress ELA Stress	Endothelial 2	*Nbl1*	0.272766651	-0.25400922	1.087642454	0.86982364
Bl Stress ELA Stress	Vascular	*Nbl1*	0.952829862	0.112288959	0.762037313	0.8692493
ELA Ctrl ELA Stress	NPCs	*Nbl1*	0.953373913	0.064056279	0.39914922	0.392016392
ELA Ctrl ELA Stress	CA3 Glut 1	*Nbl1*	2.00958E-10	-0.66886375	1.092528095	0.699924851
ELA Ctrl ELA Stress	CA3 Glut 2	*Nbl1*	1.10006E-05	-1.1160067	0.778015737	0.321384128
ELA Ctrl ELA Stress	CA3 Glut 3	*Nbl1*	2.13954E-08	-1.20071285	0.734353096	0.25781934
ELA Ctrl ELA Stress	CA1 Glut 1	*Nbl1*	1.00E-16	-0.88646449	0.619767306	0.301681546
ELA Ctrl ELA Stress	Pericytes	*Nbl1*	0.986007376	-0.00567837	1.139461906	1.173891677
ELA Ctrl ELA Stress	Endothelial 2	*Nbl1*	0.552922749	-0.20803137	0.912775269	0.86982364
ELA Ctrl ELA Stress	Vascular	*Nbl1*	0.934634449	0.174396084	0.678607903	0.8692493

Supplemental Table 1: Expression of genes selected for RNAscope validation. Mean expression values of *Rab3b*, *Adarb2* and *Nbl1* determined by scRNA-seq.
